# Effects of biotic and abiotic factors on forest biomass fractions

**DOI:** 10.1093/nsr/nwab025

**Published:** 2021-04-02

**Authors:** Renfei Chen, Jinzhi Ran, Weigang Hu, Longwei Dong, Mingfei Ji, Xin Jia, Jingli Lu, Haiyang Gong, Muhammad Aqeel, Shuran Yao, Lizhe An, Jin-Sheng He, Karl J Niklas, Jianming Deng

**Affiliations:** State Key Laboratory of Grassland Agro-Ecosystem, School of Life Sciences, Lanzhou University, Lanzhou 730000, China; State Key Laboratory of Grassland Agro-Ecosystem, School of Life Sciences, Lanzhou University, Lanzhou 730000, China; State Key Laboratory of Grassland Agro-Ecosystem, School of Life Sciences, Lanzhou University, Lanzhou 730000, China; State Key Laboratory of Grassland Agro-Ecosystem, School of Life Sciences, Lanzhou University, Lanzhou 730000, China; State Key Laboratory of Grassland Agro-Ecosystem, School of Life Sciences, Lanzhou University, Lanzhou 730000, China; Yanchi Research Station, School of Soil and Water Conservation, Beijing Forestry University, Beijing 100083, China; State Key Laboratory of Grassland Agro-Ecosystem, School of Life Sciences, Lanzhou University, Lanzhou 730000, China; State Key Laboratory of Grassland Agro-Ecosystem, School of Life Sciences, Lanzhou University, Lanzhou 730000, China; State Key Laboratory of Grassland Agro-Ecosystem, School of Life Sciences, Lanzhou University, Lanzhou 730000, China; State Key Laboratory of Grassland Agro-Ecosystem, School of Life Sciences, Lanzhou University, Lanzhou 730000, China; State Key Laboratory of Grassland Agro-Ecosystem, School of Life Sciences, Lanzhou University, Lanzhou 730000, China; State Key Laboratory of Grassland Agro-Ecosystem, School of Life Sciences, Lanzhou University, Lanzhou 730000, China; Plant Biology Section, School of Integrative Plant Science, Cornell University, Ithaca, NY 14853, USA; State Key Laboratory of Grassland Agro-Ecosystem, School of Life Sciences, Lanzhou University, Lanzhou 730000, China

**Keywords:** biomass allocation, leaf biomass, allometry, plant density, plant height, scaling exponents

## Abstract

The extent to which key factors at the global scale influence plant biomass allocation patterns remains unclear. Here, we provide a theory about how biotic and abiotic factors influence plant biomass allocation and evaluate its predictions using a large global database for forested communities. Our analyses confirm theoretical predictions that temperature, precipitation, and plant height and density jointly regulate the quotient of leaf biomass and total biomass, and that they have a much weaker effect on shoot (leaf plus stem) biomass fractions at a global scale. Moreover, biotic factors have larger effects than abiotic factors. Climatic variables act equally on shoot and root growth, and differences in plant body size and age, as well as community species composition, which vary with climate in ways that drown out the variations in biomass fractions. The theory and data presented here provide mechanistic explanations of why climate has little effect on biomass fractions.

## INTRODUCTION

How plants allocate their biomass to construct new leaves, stems, and roots is an important issue in ecology because biomass allocation patterns affect many ecological processes [1,2]. Moreover, biomass allocation patterns can change in response to abiotic and biotic factors in ways that can indirectly affect biodiversity and ecosystem function [3–5]. Therefore, it is necessary to quantitatively understand biomass allocation patterns and how they respond to biotic and abiotic variables for both theoretical and practical reasons [5].

Two approaches have been traditionally used to study biomass allocation patterns. One approach utilizes the biomass ratios or biomass fractions of the three plant organ-types (leaves, stems and roots) [5,6]. For example, the partitioning of belowground (roots) and aboveground (leaves and stems) biomass is commonly described as the root-to-shoot ratio (R/S, which is actually a quotient, not a ratio), which provides a predictor of root biomass based on the more easily measured shoot biomass [7–9]. Similarly, other biomass ratios, such as the proportion of photosynthetic to non-photosynthetic tissues, are frequently used to describe the photosynthetic capacity of forest or crop canopies [6,10]. Despite the strength of this approach, the biomass fractions of different organs are extremely sensitive and variable with respect to changes or differences in abiotic or biotic conditions. This sensitivity is reflected by numerous reports noting that root-to-leaf ratios or root-to-shoot ratios increase with increasing belowground competition for water or nutrients, whereas the same ratios decrease with increasing aboveground competition for light and space [11–13]. This variability has spurred the development of an optimal allocation theory, which predicts that plants allocate more biomass to the organ-type(s) that is (are) subject to the stronger among abiotic or biotic stresses [14]. Another concern with any ratio is that equivalent numerical values can be reached in different ways (e.g. equivalent ratios can result from decreasing denominators or increasing numerators). Therefore, although biomass fractions are mathematically simple and easily obtained, they can be unreliable or misleading as quantitative descriptors of allocation patterns across diverse species when dealing with different or changing environments.

The second classical approach is based on fitting a regression equation to describe the scaling relationship between one variable (e.g. leaf or root biomass) versus another variable (e.g. shoot or total biomass) [6,15]. The general form of this equation is *Y*_1_ = β*Y*_2_^α^, where *Y_1_* and *Y_2_* are the biomasses of any two different organ-types (e.g. leaves and stems) or biomass compartments (e.g. shoots), *β* is the normalization constant, and *α* is the scaling exponent. When this equation is log-transformed, *β* is the *y*-intercept and *α* is the slope of the log *Y*_1_ vs. log *Y*_2_ regression curve [15].

Regardless of the differences between these two approaches, a critical issue is whether they yield the same insights into how abiotic and biotic factors influence biomass allocation patterns [14]. Although early studies on biomass allocation patterns were empirically examined in the absence of theoretical underpinnings [16], more recent studies have sought (or employed) a theoretical, mechanistic underpinning, such as that found in the far-reaching metabolic scaling theory proposed by West, Brown, and Enquist (denoted henceforth as the WBE theory). This theory has provided predictions for the numerical values of the scaling exponents for a broad range of scaling relationships [17–20], and it attempts to explain why many scaling exponents are numerically ¼ or multiples of ¼ [20–25].

Arguably, the allocation patterns of photosynthetic tissues vs. non-photosynthetic tissues and leaf vs. stem (or root) biomass have attracted the most attention from plant ecologists because these patterns can be used to gauge assimilation capacities, growth rates and net primary production [6,10,12]. These patterns can also be used to estimate ecosystem carbon budgets using relatively easily measured variables of interest [7–9,26,27]. For example, using a worldwide forest database of component gross primary production fluxes, Chen *et al.* report that the allocation pattern of gross primary production is governed by both scaling constraints and three trade-offs among different components: wood production vs. fine-root production, wood production vs. leaf production, and autotrophic respiration vs. total production [28]. Nevertheless, a large gap remains in our understanding of how these scaling relationships are affected by abiotic factors such as rainfall and temperature, and the debates about these relationships are persistent. For example, Mokany *et al.* found that root-to-shoot ratios decrease with increasing precipitation, temperature, forest stand age, and plant height across different vegetation types [8], whereas Michaletz *et al.* concluded that variations in the mean annual production per individual plant are mainly driven by biotic factors such as tree age and body size and that climatic factors have little effect [29].

The aim of this paper is to further explore the constraints on plant biomass allocation from both empirical and theoretical perspectives. Herein, we present a theoretical model (see details in below) to predict the relationships for biomass fractions (for both leaves and shoots) vs. plant height (m) and biomass fractions vs. plant density (number of trees/ha), and we test these predictions using three worldwide forest biomass (kg) data sets and three different statistical methods. Further, the mechanisms are explained with both simulation and empirical approaches.

## RESULTS

### Evaluating theory using three worldwide data sets

**Figure 1.**
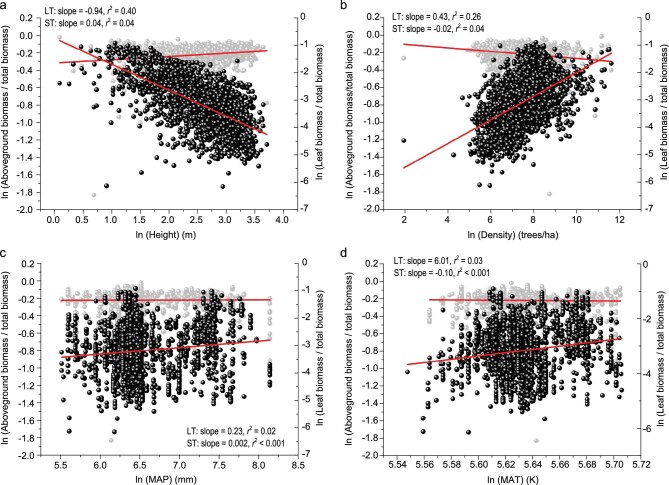
Effects of four biotic and abiotic factors on the quotient of leaf biomass and total biomass (shown in black) and the quotient of shoot biomass and total biomass (shown in grey) for forests worldwide. (a) Plant height (m), (b) plant density (number of trees/ha), (c) precipitation (mm), (d) temperature (K). The corresponding regression slopes and *r*^2^ values are provided in each plot (LT = the quotient of leaf biomass and total biomass and ST = the quotient of shoot biomass and total biomass). All variables were ln-transformed.

The numerical values of the empirically determined scaling exponents for biotic interactions (i.e. *δ, α* and *η*) were in statistical agreement with those predicted by our theoretical model for the worldwide forest data sets and different forest growth types (Table 1; also see Fig. S1 in Appendix B). Moreover, pairwise correlation analysis revealed that the quotient of leaf biomass and total biomass for all forest communities is inversely proportional to plant height (scaling exponent = −0.94; *r*^2^ = 0.40, 95% CI = −0.898; −0.992), which scaled as the 0.43 power of plant density (*r*^2^ = 0.26, 95% CI = 0.403; 0.462) but was little affected by MAP (mean annual precipitation, mm) or MAT (mean annual temperature, K) (*r*^2^ < 0.03) (Fig. 1; Table S3). Furthermore, the numerical values of the scaling exponents for the quotient of leaf biomass and total biomass with respect to both population density and plant height were statistically indistinguishable from those predicted by Eqs. 1 and 2 (Fig. 1; Table 1). As predicted by Eqs. 5 and 6, the quotient of shoot biomass and total biomass for forest communities was also insensitive to differences in plant density and plant height (*r*^2^ < 0.04) as well as MAP and MAT (*r*^2^ < 0.001) (Fig. 1).

**Table 1.** Comparison of scaling exponents determined from direct and estimated regression analysis for the relationships of the quotient of leaf biomass and total biomass vs. plant height and the quotient of leaf biomass and total biomass vs. plant density.

					Leaf biomass fraction vs. height			Leaf biomass fraction vs. density		
n		α	δ	η	Predicted	Observed^1^	Observed^2^	Predicted	Observed^1^	Observed^2^
2347	Exponent	0.77	0.89	$-$ 1.19	$-$ 0.75	$-$ 0.94	$-$ 0.81	0.36	0.43	0.10
2347	Lower 95% CI	0.756	0.871	$-$ 1.22	$-$ 0.804	$-$ 0.992	$-$ 0.842^*^	0.394	0.403	0.078^*^
2347	Upper 95% CI	0.79	0.902	$-$ 1.16	$-$ 0.676	$-$ 0.898	$-$ 0.775^*^	0.308	0.462	0.116^*^

The predicted scaling exponents of the quotient of leaf biomass and total biomass vs. height and the quotient of leaf biomass and total biomass vs. density were estimated by (2/*δ*+1)(***α***−1) and (1–1/***α***) *η* based on Eqs. 1 and 2, respectively. Where the parameters *α, δ* and *η* are the regression slopes of ln-transformed leaf biomass vs. ln-transformed total biomass, ln-transformed plant height vs. ln-transformed stem diameter and ln-transformed leaf biomass vs. ln-transformed plant density, respectively (Fig. S2). The observed^1^ scaling exponents were directly obtained from bivariate linear regression analysis, while the observed^2^ scaling exponents were directly obtained from multiple regression analysis.

**Figure 2.**
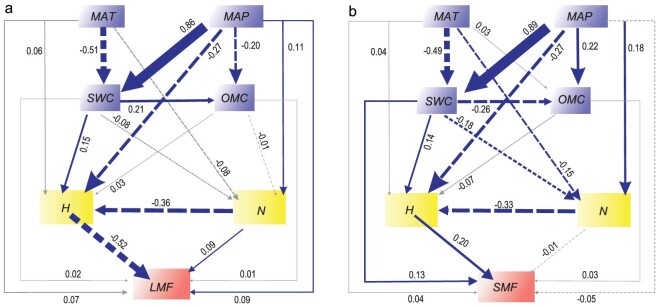
Structural equation model, depicting the effects of biotic and abiotic factors on the quotient of leaf biomass and total biomass LMF (χ^2^/df = 0.025, *P* = 0.874, RMSEA = 0.000) (a) and the quotient of shoot biomass and total biomass SMF (χ^2^/df = 0.009, *P* = 0.926, RMSEA = 0.000) (b). Plant height (*H*, m), plant density (*N*, number of trees/ha), mean annual precipitation (MAP, mm), mean annual temperature (MAT, K), soil water content (SWC) and organic matter component (OMC) explain 51.7%, 27.6%, 19.3%, 5.7%, 8.4% and 1.9% of the leaf fraction variation, respectively (20.4%, 7.1%, 4.1%, 2.9%, 17.2% and 1.5% for the shoot fraction variation, respectively). The numbers adjacent to the arrows are standardized path coefficients, which are analogous to relative regression weights, and indicative of the effect size on the relationship. Continuous and dashed arrows indicate positive and negative relationships, respectively. The width of the arrows is proportional to the strength of the path coefficients. Blue arrows denote significant relationships between two variables whereas grey arrows denote insignificant relationships. A negative relationship between plant density and plant height emerges possibly because of differences among species-specific intrinsic traits.

As predicted, multiple regression analyses (see details below) of the pooled data showed that plant height and plant density contribute the largest proportions of the variation in the quotient of leaf biomass and total biomass across all forest communities (partial *r*^2^ = 0.14, *P* < 0.001 and partial *r*^2^ = 0.007, *P* < 0.001, respectively), whereas both temperature and precipitation contributed significantly less to the variation in the quotient of leaf biomass and total biomass (partial *r*^2^ < 0.005; *P* < 0.001, *P* = 0.41, respectively) (Fig. S2; also see Table S4). In the case of the quotient of shoot biomass and total biomass, all biotic and abiotic effects were weak (Fig. S2; Table S5). The same results were observed for each of the three forest data sets (Table S6). Importantly, the scaling exponents for the quotient of leaf biomass and total biomass vs. height relationship obtained from multiple regression analysis were numerically similar to those predicted by Eq. 1 (Table 1). Similarly, structural equation models showed that biotic factors have the strongest influence on the quotients of leaf or shoot biomass and total biomass and that MAP, MAT, soil water content, and soil nutrients have little or no effect on plant height or biomass allocation (Fig. 2).

### Climate effects on the normalization constants and *RVC* (relative variation coefficient)

**Figure 3.**
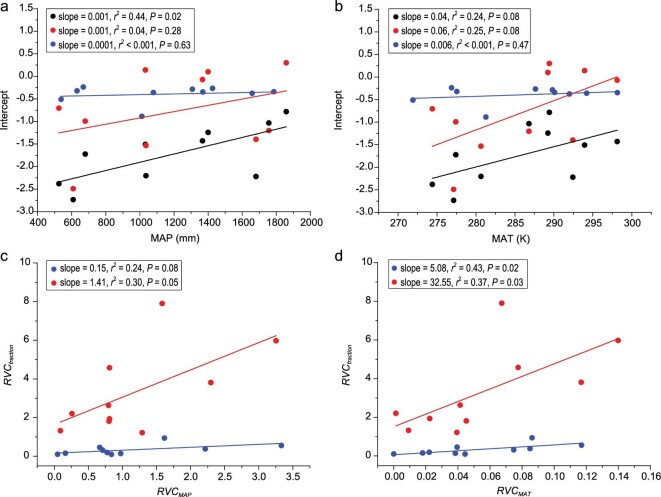
Bivariant plots and regression parameters for normalization constants vs. climate and relative biomass fraction (*RVC_fraction_* including *RVC_leaf/total_* and *RVC_shoot/total_*) vs. relative climate for forest-dwelling species in 10 plant families. (a and b) Bivariate plots of the normalization constants for the relationship between leaf vs. total biomass (data shown in black), the quotient of leaf biomass and total biomass vs. plant height (data shown in red), and the quotient of shoot biomass and total biomass vs. plant height (data shown in blue) plotted against MAP and MAT. (c and d) Regression relationships between the relative variation coefficient of precipitation (*RVC_MAP_*) or temperature (*RVC_MAT_*) and the relative variation coefficient of the quotients of leaf or shoot biomass and total biomass (data shown in red and blue, respectively). The 95% CI values of the scaling exponents are −0.073 to 2.899 and −0.026 to 0.329 for *RVC_leaf/total_* (red) and *RVC_shoot/total_* (blue) vs. *RVC_MAP_*, respectively, and 2.436 to 62.654 and 0.894 to 9.259 for *RVC_leaf/total_* and *RVC_shoot/total_* vs. *RVC_MAT_*, respectively.

The data showed that either MAP or MAT, or both, have a statistically discernible effect on the numerical values of the normalization constants for the scaling relationships of leaf vs. total biomass and the quotient of leaf biomass and total biomass vs. height (*r*^2^ = 0.44, 0.04, 0.24, 0.25) (Fig. 3a and b). This result indicates that the numerical values of the normalization constants for the scaling relationships of plant leaf biomass vs. total biomass and the quotient of leaf biomass and total biomass vs. height increase with increasing precipitation or temperature across the 10 plant families. Meanwhile, neither MAP nor MAT had a significant effect on the normalization constants for the scaling relationships of the quotient of shoot biomass and total biomass vs. height (*r*^2^ < 0.001 for both) (Fig. 3a and b).

**Figure 4.**
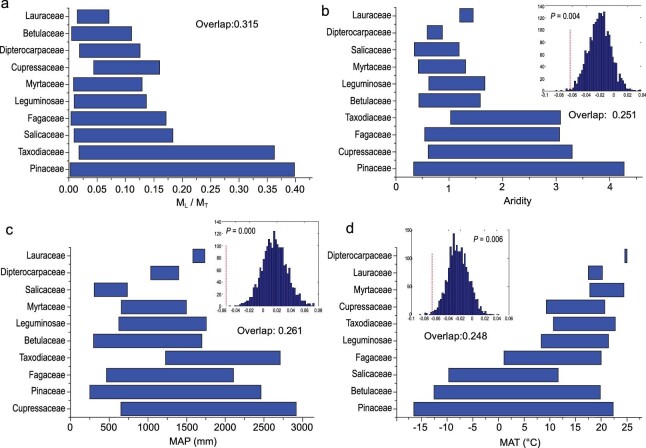
Overlap among 10 families for the quotient of leaf biomass and total biomass variation (a), climatic variables including aridity (b), MAP (c), and MAT (d). The range of the overlap for each family is significantly broader than that for each of the climatic variables (*P* ≤ 0.006) based on Monte Carlo simulations.

As predicted by Eqs. 7 and 8, both *RVC_leaf/total_* and *RVC_shoot/total_* (i.e. the *RVC* of the quotients of leaf or shoot biomass and total biomass, respectively) were significantly proportional to *RVC_MAI_* (i.e. the *RVC* of precipitation or temperature) across all of the 10 plant families (Fig. 3c and d), despite the quotients of leaf or shoot biomass and total biomass being very weakly correlated with temperature and precipitation (Figs 1, S2, S3; Tables S4, S5). Monte Carlo simulations revealed that the overlap in the numerical values of biomass fractions across the 10 families was significantly higher (0.315) than the overlap for climatic factors, including aridity (0.251, *P* = 0.004), precipitation (0.261, *P* = 0.000) and temperature (0.248, *P* = 0.006) (Fig. 4). The quotients of shoot biomass and total biomass conformed to the same overall trend.

## DISCUSSION

One of the most important challenges in ecology is to understand how plants allocate biomass to their different organ-types (i.e. leaves, stems, roots and reproductive structures). To address this challenge, scaling equations and biomass fraction methods have been used to investigate the effects of various factors on biomass allocation, and both have been used to develop different ecological theories [5,6,11,22]. Nevertheless, debates revolving around this challenge persist, and the extent to which abiotic or biotic factors at the worldwide level influence biomass allocation patterns remains unclear. One of the main reasons for this uncertainty is that vigorous theoretical underpinnings and the data sets required to test their assumptions and validity remain problematic or entirely absent. Toward this purpose, we developed a theoretical framework to elucidate how plant biomass allocation patterns are regulated by plant density and height, as well as precipitation and temperature. One of the most important predictions of this framework is the prediction that biotic factors have a larger effect on biomass fractions than abiotic factors. This prediction is supported by our empirical analysis of three worldwide forest data sets (using three different analytical protocols), which highlights the important role that intrinsic biological constraints play in influencing plant biomass allocation patterns. Additionally, we have shown that community species composition (i.e. species richness) varies with climate in ways that drown out variations in biomass fractions. As a result, climate has little effect on biomass fractions. Thus, the theory presented here provides a deeper insight into understanding the debates on the issue of how climatic factors regulate NPP (net primary production, g m^−2^ yr^−1^) [29].

As predicted, mean annual temperature and precipitation have little to no statistically discernible effect on the quotient of leaf biomass and total biomass or the quotient of shoot biomass and total biomass (Figs. 1, S2, S3). These results partially support previous conclusions about the effects of climatic variables on biomass fractions. For example, Poorter *et al.* [5] show that plants subjected to modest water stress (not severe drought) only slightly increase their root biomass fraction, and the quotient of leaf biomass and total biomass hardly changes, whereas low temperatures decrease the quotient of leaf biomass and total biomass and increase the quotient of root biomass and total biomass [5]. Recently, Reich *et al.* [30] reported that the quotients of leaf biomass and total biomass are more sensitive to variations in temperature than the quotients of root biomass and total biomass, especially for small trees (i.e. stem biomass <50 Mg/ha) [30]. These authors also reported that the largest proportion of total biomass is allocated to photosynthetic tissues in warmer environments, whereas a larger proportion of biomass is allocated to root growth in regions experiencing cold stress. Our theoretical framework and empirical evidence show that two biotic factors (height and density) are the primary determinants of the quotients of leaf biomass and total biomass for forest communities (Figs 1, S2, S3; Table S4). These findings support the notion that biomass allocation patterns can be viewed as adaptive ‘strategies’ to cope with variations in internal and external conditions. Indeed, the mechanism responsible for increasing or decreasing the quotients of leaf biomass and total biomass is a function of plant height, as disproportionally more biomass needs to be allocated to the non-photosynthetic compartments to contend with the force of gravity. An attending phenomenon is a more or less constant leaf area index despite increasing canopy height and plant density, which may optimize the interception of light [31,32]. Likewise, as predicted by Eqs. 5 and 6, the quotients of shoot biomass and total biomass converge on similar numerical values across a broad range of plant size and climate gradients (Fig. 1). We attribute the insensitivity of these quotients to climatic variables to two general factors.

First, biomass fractions tend to converge numerically on a comparatively small range of values (within the range of 0–1), whereas temperature and precipitation have a large continuous range along environmental or geographic gradients at the global scale. This result suggests to us that the structure or taxonomic composition of a plant community will likely shift along steep environmental gradients (e.g. temperature with latitude) at the global scale, whereas the numerical values of the quotients of leaf and total biomass are restricted to a smaller range for each plant family at the local level (Fig. 4). In addition, variations in the quotients of leaf or shoot biomass and total biomass are significantly influenced by the extent to which local environments impose stress on vegetation (e.g. *RVC_MAI_*, Fig. 3). Nevertheless, although differences in climatic variables at larger scales can predict, to some degree, species richness, our results show that they cannot predict variations in biomass allocation patterns over large geographic scales. The reason is that changes in species richness over large geographic scales overwhelm species-specific morphometric plasticity within the geographic range of any given species (see Fig. 4). Additionally, as predicted by our models, climatic factors usually act simultaneously on the shoots and roots of a plant such that the effect of climatic factors on biomass fractions would be considerably offset by the ratio of leaf or shoot biomass to total biomass.

Second, local abiotic environmental conditions in most forested communities are generally not sufficiently stressful to alter what appear to be the intrinsically optimal biomass allocation patterns of most forest-dwelling species. To explore this proposition further, we performed additional analyses using data for soil water content and the Priestley-Taylor alpha coefficient, which are considered effective in describing overall aridity stress on vegetation. These analyses indicate that both of these soil water balance variables (soil water content and the Priestley-Taylor alpha coefficient) have no strong effect on the quotients of leaf or shoot biomass and total biomass (Fig. S4; Fig. 2). In addition, soil nutrients (organic carbon and total nitrogen) have little effect on either of the two quotients (Fig. 2; Table S9). In contrast, biotic factors, such as tree height and density, impose significantly more biotic constraints and contribute most to the variations observed in the quotients of leaf or shoot biomass and total biomass. The strong effect of biotic factors on the quotient of leaf biomass and total biomass is consistent with the predictions from metabolic scaling theory, which predicts scaling exponents ranging from 1/1 for small plants to 3/4 for large trees [32–36]. This variation indicates that the quotient of leaf biomass and total biomass decreases with increasing overall biomass, which correlates with increasing plant height and girth. This trend is often considered to be the result of the accumulation of secondary dead tissues such as wood [15,25]. Likewise, growth in height is considered to be limited by hydraulic transport limitations, which constrain leaf biomass and the quotients of leaf biomass and total biomass [37,38]. Note that plant height has no statistically strong relationship with precipitation and temperature (Fig. S5). In addition, the weak climate effect on plant biomass may emerge from differences between NPP and standing biomass, and differences between plant size and age (Appendix C). The relatively low spatial resolution of soil and climatic variables in the data sets used in our study may also contribute to the statistically weak associations between these abiotic and biomass variables.

The aforementioned conclusions for plant biomass are consistent with some but not all prior empirical studies. For example, Michaletz *et al.* suggest that variation in the NPP of woody plants results mainly from biotic factors with respect to plant age and total biomass, as opposed to climatic factors such as mean annual temperature and precipitation [29,39], whereas Chu *et al.* argue that climate has a direct influence on NPP [40]. We believe that this difference of opinion may rest in part on the statistical criteria used to evaluate scaling relationships. For example, in our study, pairwise correlation and regression analyses show that the leaf fraction is, in fact, tightly related to MAT and MAP, but the explanatory power (i.e. *r*^2^) of these relationships is extremely poor compared with that for the biotic factors (Fig. 1). Furthermore, multiple regression analyses similarly show that biotic factors are considerably better predictors of the quotient of leaf biomass and total biomass than abiotic factors (Fig. S2). However, the correlations of the quotient of shoot biomass and total biomass versus both the abiotic and biotic factors are much weaker compared with the quotient of leaf biomass and total biomass, which is consistent with the reports by Cairns *et al.* (1997) and Wang *et al.* (2008) [7,41]. This phenomenon appears to be an emergent feature of the fact that aboveground plant biomass scales, on average, isometrically with respect to belowground biomass [1,9,22], such that the root-to-shoot ratio remains approximately constant regardless of species differences. Indeed, our model and the analysis presented here demonstrate that this relationship may be a canonical feature of plant communities that are under no serious environmental stress.

We also found that the intercepts for the scaling relationships of plant leaf biomass vs. total biomass, and the quotient of leaf biomass and total biomass vs. plant height significantly increase with increasing precipitation and temperature for the pooled data across 10 plant families (Fig. 3). The significantly positive correlations between the normalization constants vs. the scaling exponents for the scaling relationships of leaf biomass vs. total biomass indicate that leaf biomass per plant increases faster with respect to increasing total biomass or plant size in mild environments characterized by equitable temperatures and high rainfall (e.g. tropical forests) than in harsh environments (e.g. arid or high elevation communities). This relatively rapid increase in leaf biomass per plant among trees growing in mild environments is likely the result of relatively greater access to light resources and the faster regeneration of foliage compared to plants growing in harsher environments. In contrast, the strongly negative correlations between the normalization constants vs. the scaling exponents for the scaling relationships of the quotient of leaf biomass and total biomass vs. height suggest that the quotient of leaf biomass and total biomass per plant declines faster with increasing plant height in mild environments than that in harsh environments. Our model predicts (and the three analytical approaches presented here confirm) that biotic factors (e.g. plant height) and interactions (i.e. plant density) have a more significant and important effect on biomass allocation patterns than abiotic factors (e.g. rainfall and temperature). Our study shows that the biomass allocation patterns of trees are invariant over a broad range of environmental conditions, but vary with plant intrinsic characteristics. Future work must focus on comparable data sets (and modes of analyses) for diverse plant communities including herbs and shrubs growing under ecologically stressful conditions. Our model indicates that if these forest communities are well adapted to these conditions, results similar to those reported here will be forthcoming. Our model also predicts that the imposition of stress on communities previously adapted to local environmental conditions, such as stress incurred by climate change, will have discernible effects on biomass allocation conditions. The theoretical framework presented here can be used to estimate the leaf biomass fractions of trees based on environmental factors and other plant traits and can provide a foundation to simultaneously (rather than separately) test optimal and allometric allocation theory.

### A theoretical framework for biomass allocation

Our theoretical framework for predicting variations in standing leaf and shoot (i.e., the sum of leaves and stems) biomass fractions employs previously confirmed scaling relationships for plant traits and general equations derived from the metabolic scaling theory [17–20].

The construction of our theory begins by recognizing that the scaling exponents of standing leaf biomass per plant (*M_L_*) vs. whole plant biomass (*M_T_*) and of average height per plant (*H*) vs. average stem diameter per plant (*D*) are reported to be more or less invariant across species [15,17]. Moreover, these exponents take the general form of ${Y_1} \propto {Y_2}^\alpha $ (where *Y*_1_ and *Y*_2_ are any two interdependent variables of interest, and *α* is a scaling exponent), i.e., ${M_L} \propto {M_T}^\alpha $ and $H \propto {D^\delta }$ (where *δ* is another scaling exponent). Thus, it follows that the quotient of leaf biomass and total biomass can be expressed as $\frac{{{M_L}}}{{{M_T}}} \propto \frac{{M_T^\alpha }}{{{M_T}}} \propto M_T^{\alpha - 1}$. It also follows that ${M_T} \propto {D^2}H$, such that *M_T_* can be expressed generically as ${M_T} \propto {D^2}H \propto {H^{(2/\delta ) + 1}}$, and the quotient of leaf biomass and total biomass takes the general form

(1) \begin{equation*}\frac{{{M_L}}}{{M{}_T}} = {\beta _1}{H^{(2/\delta + 1)(\alpha - 1)}}\end{equation*}

where *β*_1_ is a normalization constant.

Previous work has also shown that leaf biomass per individual plant declines inversely with increasing population density (*N*), such that ${M_L} \propto {N^{ - 1}}$ [31–33,42]. Therefore, it is reasonable to assume that the relationship between leaf biomass and plant density takes the form ${M_L} \propto {N^\eta }$, where *η* is another scaling exponent that depends on the extent of canopy crowding.

Given the preceding generalizations, for any specified plant population, the quotient of leaf biomass and total biomass per plant can be expressed as

(2) \begin{equation*}\frac{{{M_L}}}{{{M_T}}} = {\beta _2}{N^{\eta (\alpha - 1)/\alpha }}\end{equation*}

where *β*_2_ is another constant. Because temperature (*T*) and precipitation (*P*) affect plant growth and net primary productivity (29, 39, 40), we assume that temperature and precipitation do not act equally on the growth rate of leaves (or shoots) and roots such that the equations for *M_L_* and *M_T_* must be modified to incorporate the combined effects of these two variables. Moreover, we assume that plant biomass is characterized by a power-law dependence on temperature and precipitation, and the effects of temperature and precipitation are multiplicative (29). Specifically, ${{{M}}_{{L}}} \propto {{{P}}^{{{{\varepsilon }}_{{1}}}}}{{{T}}^{{{{\theta }}_{{1}}}}}$ and ${{{M}}_{{T}}} \propto {{{P}}^{{{{\varepsilon }}_{{2}}}}}{{{T}}^{{{{\theta }}_{{2}}}}}$, where *ϵ*_1_ and *ϵ*_2_ are scaling exponents for leaf biomass and total biomass with respect to precipitation, and ${\rm{\theta }}$_1_ and ${\rm{\theta }}$_2_ are scaling exponents for leaf biomass and total biomass with respect to temperature. Thus, the overall equation for the leaf biomass fraction becomes

(3) \begin{equation*}\frac{{{{{M}}_{{L}}}}}{{{{{M}}_{{T}}}}}= {{\rm{\beta }}_{\rm{3}}}{{{P}}^{{\tau }}}{{{T}}^{{\omega }}}{{{H}}^{{\rm{(2/\delta + 1)(\alpha - 1)}}}}{{{N}}^{{\rm{(}}\frac{{{\rm{\alpha - 1}}}}{{\rm{\alpha }}}{\rm{)\eta }}}},\end{equation*}

where *β*_3_ is also a normalization constant, ${\rm{\tau }}={{\rm{\varepsilon }}_{\rm{1}}}{\rm{ - }}{{\rm{\varepsilon }}_{\rm{2}}}$, and ${\rm{\omega }}={{\rm{\theta }}_{\rm{1}}}{\rm{ - }}{{\rm{\theta }}_{\rm{2}}}$ (whose numerical values are determined by abiotic factors). Equation (3) can be linearized as

(4) \begin{eqnarray*}{{\rm ln}}\left(\frac{{{{{M}}_{{L}}}}}{{{{{M}}_{{T}}}}}\right) &=& {\rm{ln}}\left({{\rm{\beta }}_{\rm{3}}}\right) + \tau {\rm ln}(P)+ \omega\, {\rm ln}(T)\nonumber\\ &&+ (2/\delta + 1)(\alpha - 1){\rm In}(H) \nonumber\\&&+ \left(\frac{{{\rm{\alpha - 1}}}}{{\rm{\alpha }}}\right){{\eta {\rm ln}(N)}}.\end{eqnarray*}

Previous studies also indicate that there is an isometric scaling relationship between shoot biomass (*M*_S_) and root biomass (*M*_R_), such that ${{{M}}_{{S}}}={{\rm{\beta }}_{\rm{4}}}{{M}}_{{R}}^{{1}}$, where *β*_4_ is another normalization constant that depends on the characteristics of a species and its local environmental conditions [1,13,22]. Therefore, the quotient of shoot biomass and total biomass is predicted to be constant. That is, ${{{M}}_{{S}}}{\rm{/}}{{{M}}_{{T}}}={{\rm{\beta }}_{\rm{4}}}{\rm{/(1 }}+{{\rm{\beta }}_{\rm{4}}})={{\rm{\beta }}_{\rm{4}}}{\rm{/(1}}+{{\rm{\beta }}_{\rm{4}}}{{)M}}_{{T}}^{{0}}$.

The relationship between total biomass and plant height yields two important conclusions: (1) the quotient of shoot biomass and total biomass is invariant with respect to plant height, and (2) the quotient of shoot biomass and total biomass is invariant with respect to plant density:

(5) \begin{equation*}\frac{{{{{M}}_{{S}}}}}{{{{{M}}_{{T}}}}}=\frac{{{{\rm{\beta }}_{{4}}}}}{{{\rm{1}}+{{\rm{\beta }}_{\rm{4}}}}}{{{H}}^{\rm{0}}}\end{equation*}

(6) \begin{equation*}\frac{{{{{M}}_{{S}}}}}{{{{{M}}_{{T}}}}}=\frac{{{{\rm{\beta }}_{\rm{4}}}}}{{{\rm{1}}+{{\rm{\beta }}_{\rm{4}}}}}{{{N}}^{\rm{0}}}\end{equation*}

### Abiotic factors

As different plant taxa or floras have different biogeographic distribution ranges and different ecological niche breadths, species distribution patterns usually vary as a function of the climatic variations across biogeographical gradients. Accordingly, changes in plant species along environmental gradients are likely, at least in part, to overwhelm the variations in phenotypic traits, such as the biomass fractions for a given species within its distribution range. In other words, species richness or community structure can continuously vary with climatic variations at the global scale, whereas the response “plasticity” of any functional trait for any given species is likely limited within the distribution range of the particular species. In this case, it is important to explore how the phenotypic plasticity of biomass fractions varies in response to climatic variables within the natural distribution range of a given plant species rather than across the global biogeographical scale.

To this end, we explored how local climatic variables affect biomass allocation patterns along climatic gradients by noting that the extent to which climatic variables induce plastic responses to stress can be described by the relative variation coefficient (*RVC*) of a climatic variable. This parameter is given by the formula ${\mathit {RVC}}_{\mathit {MAI}} = \frac{\mathit{MAI}_{\max}\,-\, {\mathit{MAI}}_{\min}}{{\mathit{MAI}}_{\rm{average}}}$, where *RVC_M__AI_* is the *RVC* of precipitation or temperature (i.e., *RVC_MAP_* or *RVC_MAT_*) and *MAI*_max_, *MAI*_min_, and *MAI*_average_ are the maximum, minimum, and average values, respectively, of annual mean precipitation or temperature within the distribution range of any given plant species or family. We hypothesized that the *RVC* of the quotients of leaf or shoot biomass and total biomass for any given plant taxon is determined by abiotic factors, which can be evaluated in the context of the climatic variables that impose the greatest stress on a particular taxon. Since all the values of *RVC* (*RVC_leaf__/total_, RVC_shoot__/total_, RVC_MAI_*) can vary between only 0 and 1, log-transformation of the raw data has little effect on the relationship between dependent and independent variables. Thus, we hypothesized that *RVC_leaf__/total_* vs. *RVC_MAI_* and *RVC_shoot__/total_* vs. *RVC_MAI_* conform to linear relationships. If true, the *RVC*s of the biomass fractions for any taxon can be expressed as

(7) \begin{equation*}{\mathit {RVC}}_{\scriptsize\textit {leaf{/}total}} = {\beta _{{5}}}{\mathit {RVC}}_{\mathit {MAI}}\end{equation*}

and

(8) \begin{equation*}\quad \quad \quad {\mathit {RVC}}_{\scriptsize\textit {shoot{/}total}}= {\beta _6}{\mathit {RVC}}_{\mathit {MAI}}\end{equation*}

where *RVC_leaf__/total_* and *RVC_shoot__/total_* equal the range of the variation in the quotients of leaf or shoot biomass and total biomass, respectively, within the geographic distribution of a taxon divided by the mean value of all local quotients of leaf or shoot biomass and total biomass. Once again, *β*_5_ and *β*_6_ are normalization constants.

## Supplementary Material

nwab025_Online_Appendixs
