# Supplementary material for: Effects of biotic and abiotic factors on forest biomass fractions
Source: Natl Sci Rev. 2021 Apr 2;8(10):nwab025. doi: 10.1093/nsr/nwab025 (PMC8566188; doi:10.1093/nsr/nwab025)
Supplement: nwab025_Online_Appendixs [file nwab025_online_appendixs.zip › AppendixA.docx]

**Materials and methods**

**A theoretical framework for biomass allocation**

Our theoretical framework for predicting variations in standing leaf and shoot (i.e., the sum of leaves and stems) biomass fractions employs previously confirmed scaling relationships for plant traits and general equations derived from the metabolic scaling theory [17-20].

The construction of our theory begins by recognizing that the scaling exponents of standing leaf biomass per plant (*ML*) vs. whole plant biomass (*MT*) and of average height per plant (*H*) vs. average stem diameter per plant (*D*) are reported to be more or less invariant across species [15,17]. Moreover, these exponents take the general form of (where *Y*1 and *Y*2 are any two interdependent variables of interest, and *α* is a scaling exponent), i.e., and (where *δ* is another scaling exponent). Thus, it follows that the quotient of leaf biomass and total biomass can be expressed as . It also follows that , such that *MT* can be expressed generically as , and the quotient of leaf biomass and total biomass takes the general form

(1)

where *β*1 is a normalization constant.

Previous work has also shown that leaf biomass per individual plant declines inversely with increasing population density (*N*), such that [31-33, 42]. Therefore, it is reasonable to assume that the relationship between leaf biomass and plant density takes the form , where *η* is another scaling exponent that depends on the extent of canopy crowding.

Given the preceding generalizations, for any specified plant population, the quotient of leaf biomass and total biomass per plant can be expressed as

(2)

where *β*2 is another constant. Because temperature (*T*) and precipitation (*P*) affect plant growth and net primary productivity (29, 39, 40), we assume that temperature and precipitation do not act equally on the growth rate of leaves (or shoots) and roots such that the equations for *ML* and *MT* must be modified to incorporate the combined effects of these two variables. Moreover, we assume that plant biomass is characterized by a power-law dependence on temperature and precipitation, and the effects of temperature and precipitation are multiplicative (29). Specifically, and , where *ε*1 and *ε*2 are scaling exponentsfor leaf biomass and total biomass with respect to precipitation, and 1 and 2 are scaling exponentsfor leaf biomass and total biomass with respect to temperature. Thus, the overall equation for the leaf biomass fraction becomes

, (3)

where *b*3 is also a normalization constant, , and (whose numerical values are determined by abiotic factors). Equation (3) can be linearized as

, (4)

Previous studies also indicate that there is an isometric scaling relationship between shoot biomass (*M*S) and root biomass (*M*R), such that , where *β*4 is another normalization constant that depends on the characteristics of a species and its local environmental conditions [1, 13, 22]. Therefore, the quotient of shoot biomass and total biomass is predicted to be constant. That is, .

The relationship between total biomass and plant height yields two important conclusions: (1) the quotient of shoot biomass and total biomass is invariant with respect to plant height, and (2) the quotient of shoot biomass and total biomass is invariant with respect to plant density:

(5)

(6)

**Abiotic factors**

As different plant taxa or floras have different biogeographic distribution ranges and different ecological niche breadths, species distribution patterns usually vary as a function of the climatic variations across biogeographical gradients. Accordingly, changes in plant species along environmental gradients are likely, at least in part, to overwhelm the variations in phenotypic traits, such as the biomass fractions for a given species within its distribution range. In other words, species richness or community structure can continuously vary with climatic variations at the global scale, whereas the response “plasticity” of any functional trait for any given species is likely limited within the distribution range of the particular species. In this case, it is important to explore how the phenotypic plasticity of biomass fractions varies in response to climatic variables within the natural distribution range of a given plant species rather than across the global biogeographical scale.

To this end, we explored how local climatic variables affect biomass allocation patterns along climatic gradients by noting that the extent to which climatic variables induce plastic responses to stress can be described by the relative variation coefficient (*RVC*) of a climatic variable. This parameter is given by the formula , where *RVCMAI* is the *RVC* of precipitation or temperature (i.e., *RVCMAP* or *RVCMAT*) and *MAI*max, *MAI*min, and *MAI*average are the maximum, minimum, and average values, respectively, of annual mean precipitation or temperature within the distribution range of any given plant species or family. We hypothesized that the *RVC* of the quotients of leaf or shoot biomass and total biomass for any given plant taxon is determined by abiotic factors, which can be evaluated in the context of the climatic variables that impose the greatest stress on a particular taxon. Since all the values of *RVC* (*RVCleaf/total*, *RVCshoot/total*, *RVCMAI*) can vary between only 0 and 1, log-transformation of the raw data has little effect on the relationship between dependent and independent variables. Thus, we hypothesized that *RVCleaf/total* vs. *RVCMAI* and *RVCshoot/total* vs. *RVCMAI* conform to linear relationships. If true, the *RVC*s of the biomass fractions for any taxon can be expressed as

*RVCleaf/total = β5 RVCMAI* (7)

and *RVCshoot/total = β6 RVCMAI* (8)

where *RVCleaf/total* and *RVCshoot/total* equal the range of the variation in the quotients of leaf or shoot biomass and total biomass, respectively, within the geographic distribution of a taxon divided by the mean value of all local quotients of leaf or shoot biomass and total biomass. Once again, *β*5 and *β*6 are normalization constants.

**Data sets**

Three forest data sets were used to examine the predictions of our theoretical framework. Each of these data sets was vetted by a stringent review process, and each data set has been widely used by ecologists as a research tool to examine biomass allocation patterns [30]. The three data sets are (1) the worldwide compilation of Cannell (1982) [43], (2) the forest biomass and primary production compendium for Eurasian species compiled by Usoltsev (2001) [44], and (3) the primary literature on Chinese forests compiled by Luo *et al.* (2014) [45]. The three data sets collectively reflect the characteristics of 2347 forest communities distributed worldwide and span a broad range of precipitation and temperature conditions (figure 1).

The data from these three compilations were culled using two criteria: i) the data reported for each forest community must contain all of the variables required to test our model (i.e., leaf biomass, root biomass, shoot biomass, total biomass, height, basal stem diameter, and plant density). Tree-level biomass (oven-dried mass) was measured by destructive harvesting and by drying and weighing the various biomass components (also see Cannell (1982) and Luo *et al.* (2014) for details), and ii) all of the variables must have been directly measured and not estimated (i.e., data estimated by means of allometric equations were rejected for analyses). For example, 6184 out of the 8033 forest community data sets compiled by Usoltsev were excluded because some variables were not measured directly or were estimated by allometric equations. Likewise, only 349 out of 1602 reports on the forest communities compiled by Luo *et al.* (2014) met the two criteria used in our study.

Using the geographic data reported for each forest community, the data on mean annual precipitation (MAP) and mean annual temperature (MAT) were retrieved from the <http://wcatlas.iwmi.org/> website, and the data on the fraction of soil water content and Priestley-Taylor alpha coefficient were retrieved from the <http://www.cgiar-csi.org/data/global-high-resolution-soil-water-balance> website. The aridity index (AI) was calculated by the ratio of precipitation to potential evapotranspiration. Five parts of aridity were identified based on this standard: humid regions (with AI values larger than 0.65); dry, subhumid regions (with AI values from 0.5 to 0.65); semi-arid regions (with AI values from 0.2 to 0.5); arid regions (with AI values from 0.05 to 0.2) and hyperarid regions (with AI values smaller than 0.05).

**Statistical analysis**

Bivariate regression analyses were used to determine the scaling relationships for leaf biomass vs. total biomass, plant height vs. basal diameter, and leaf biomass vs. plant density. The effects of plant height and density (biotic factors) and MAP and MAT (abiotic factors) on the quotients of leaf or shoot biomass and total biomass were also analysed using bivariate regression protocols [46]. Similarly, the relationships between the numerical values of the scaling exponents and intercepts were analysed. All biomass variables were at the individual plant level.

To test whether the scaling relationships predicted by Eqs. 1-6 were consistent with the empirical data, multiple regression analyses (using software R) were used to determine the effects of biotic and abiotic factors on the biomass fractions of different organs. These equations were also used to determine whether biotic or abiotic factors contributed the most to the variations in the quotient of leaf biomass and total biomass. Partial regression plots were acquired from the avPlot function in the R package.All variables employed in multiple regression analyses were independent (i.e., they manifested no collinearity) based on variance inflation factor (VIF) values, which did not exceed the threshold value of 10 [29]. The VIF values for each covariate were calculated for the candidate models. Each value was less than 3, with the exception of total biomass (table S1). To select the best model for multiple regression analysis, the Akaike information criterion (AIC) was used after calculating the VIF values for each covariate in each model [29, 47].

The AIC values were calculated for all possible models consisting of candidate independent variables (i.e., plant height, plant density, MAP, and MAT). The model with the best AIC value was then selected to describe the scaling relationships for the quotient of leaf biomass and total biomass versus multiple biotic/abiotic factors based on the criteria of the lowest ΔAIC and AIC values [48, 49]. The selection of the model with the best AIC value and all related analyses were performed in the R environment. The selected models with the best AIC values indicated that the predictions of the effects on biomass fractions should include both biotic and abiotic factors (ΔAIC = 0, AIC = 4819.319; also see table S2). Furthermore, structural equation models in Amos 21.0 (IBM, Chicago, USA) were constructed to analyse the direct and indirect influence of climate variables on biomass allocation. Standardized path coefficients were calculated with the maximum likelihood method to evaluate the relative importance of temperature or precipitation as well as soil water content and soil nutrients effects on plant leaf fraction or shoot fraction directly or indirectly through plant height and density. With the standardized path coefficients presented in figure 3, the total effects of climate variables and soil property on plant biomass allocation were predicted by summing the indirect (the product of all coefficients in one pathway) and direct standardized path coefficients. Note that soil nutrients are represented by organic matter component (OMC) which is the first component from a PCA conducted with soil organic Carbon and total Nitrogen, and specific methods can be seen in reference 50.

A large overlap in biomass allocation traits among forest communities is expected if allocation patterns do not vary along climatic gradients. Accordingly, we used Monte Carlo simulations to test whether the degrees of overlap in allocation traits among communities are significantly greater than those in climatic variables. For this purpose, we sorted the dataset into ten dominant plant families (sample size is larger than 5 for each family), i.e., *Betulaceae*, *Cupressaceae*, *Dipterocarpaceae*, *Fagaceae*, *Lauraceae*, *Leguminosae*, *Myrtaceae*, *Pinaceae*, *Salicaceae*, and *Taxodiaceae*. Each family has distinct maximum and minimum values (range = maximum - minimum) for all given biotic and abiotic variables. The degree of overlap was then calculated as the ratio of the sum of pairwise overlaps to the sum of the ranges over all pairs. To compare the degree of overlap between a climatic variable and an allocation trait, Monte Carlo simulations were run by randomly placing the position of the range of the climatic variable for each family within the overall range across the ten families. Similar randomizations were also applied to the allocation trait. For each simulation, we calculated the difference in the degree of overlap between the climatic variable and the allocation trait. A null distribution could then be established by repeating the simulation 2000 times. Finally, the significance of the observed difference in the degree of overlap could be tested against the null distribution.

Plant net primary production (NPP) is obviously different from plant biomass, although they can exhibit a strong correlation. Therefore, we used similar statistical methods to examine the effect of climate on NPP for the different organ compartments (i.e., leaf NPP, total NPP, aboveground NPP and belowground NPP) as well as the leaf NPP fraction (i.e., leaf NPP/total NPP) and the shoot NPP fraction (i.e., aboveground NPP/total NPP) using the Usoltsev (2001) data set (the other two data sets provided no information about NPP). To explain the mechanism of the effect of climate, we analysed the effect of climate on plant biomass for different age classes.

1. Niklas KJ, Midgley JJ, Enquist BJ. 2003 A general model for mass–growth–density relations across tree–dominated communities. *Evol. Ecol. Res.* **5**, 459–468. (<http://www.evolutionary-ecology.com/abstracts/v05/1524.html>)
2. Cannell MGR. 1982 World forest biomass and primary production data. New York: Academic Press.
3. Usoltsev VA. 2001 Forest Biomass of Northern Eurasia. Ural Branch: Russian Academy of Sciences, Botanical Garden and Ministry of Education of Russian Federation, Ural State Forest Engineering University.
4. Luo Y, Zhang X, Wang X, Lu F. 2014 Biomass and its allocation of Chinese forest ecosystems. *Ecology* **95**, 2026–2026. ([https://doi.org/ 10.1890/13-2089.1](https://doi.org/%2010.1890/13-2089.1))
5. Tian D *et al.* 2018 Global leaf nitrogen and phosphorus stoichiometry and their scaling exponent. *Natl Sci Rev* **5**:728–739.
6. Ryan TP. 1997 Modern Regression Methods. Wiley.
7. Wagenmakers EJ, Farrell S. 2004 AIC model selection using Akaike weights. *Psychon B Rev*. **11**, 192-196. ([https://link.springer.com/article/10.3758/ BF03206482](https://link.springer.com/article/10.3758/%20BF03206482))
8. Ji MF, Deng JM, Yao BQ, Chen RF, Fan ZX, Guan JW, Li XW, Wu F, Niklas KJ. 2016 Ecogeographical variation of 12 morphological traits within *Pinus tabulaeformis*: The effects of environmental factors and demographic histories. *J. Plant Ecol.* rtw033. (https://doi.org /10.1093/jpe/rtw033)
9. Delgado-Baquerizo M *et al.* 2013 Decoupling of soil nutrient cycles as a function of aridity in global drylands. *Nature* **502**, 672-676.
