# Supplementary material for: Effects of biotic and abiotic factors on forest biomass fractions
Source: Natl Sci Rev. 2021 Apr 2;8(10):nwab025. doi: 10.1093/nsr/nwab025 (PMC8566188; doi:10.1093/nsr/nwab025)
Supplement: nwab025_Online_Appendixs [file nwab025_online_appendixs.zip › AppendixB.docx]

**Supporting Information**


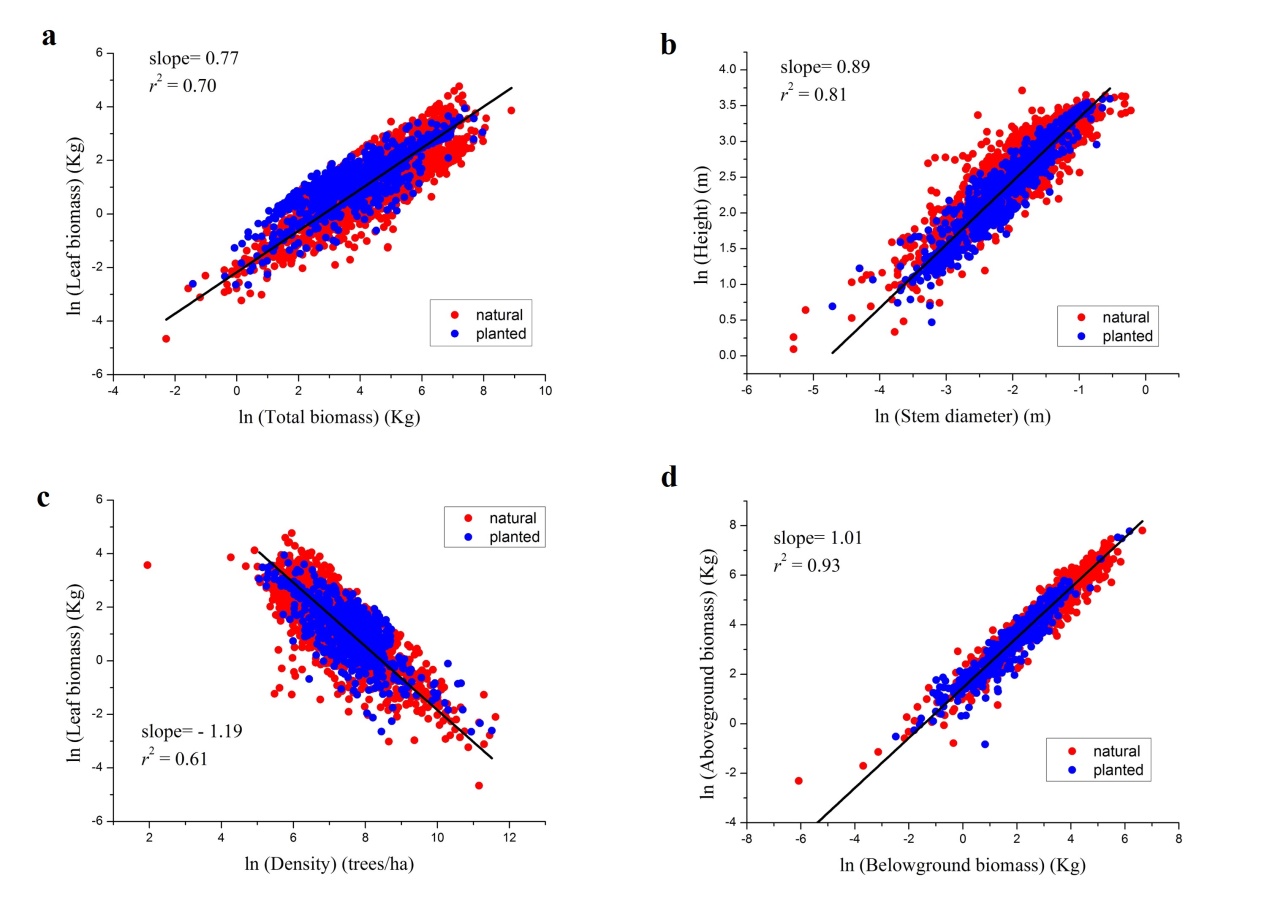


**Figure S1.** **Log-log bivariate relationships among variables of interest drawn from all forest data sets. Different colours denote different forest growth types (red = all trees of natural forests; blue = all trees of planted forests).** All regressions are significant at *P* < 0.0001. **(a)** Total biomass per plant (Kg) vs. leaf biomass per plant (Kg). **(b)** Plant height (m) vs. basal stem diameter (m). **(c)** Plant density (trees/ha) vs. leaf biomass per plant (Kg). (**d**) Aboveground biomass per plant (Kg) vs. belowground biomass per plant (Kg).


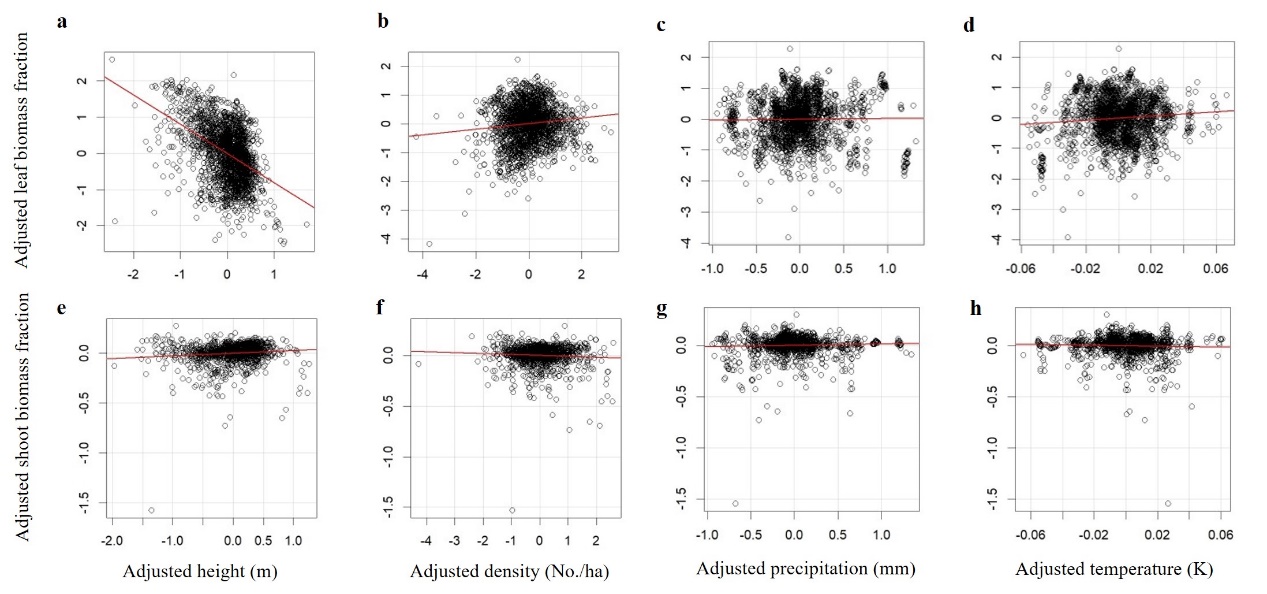


**Figure S2.** **Partial regression relationships between biomass fraction and four influencing factors for forests worldwide. (a-d)** Relationships of the quotient of leaf biomass and total biomass vs. plant height, the quotient of leaf biomass and total biomass vs. plant density, the quotient of leaf biomass and total biomass vs. precipitation, and the quotient of leaf biomass and total biomass vs. temperature, respectively. **(e-h)** Relationships of the quotient of shoot biomass and total biomass vs. plant height, the quotient of shoot biomass and total biomass vs. plant density, the quotient of shoot biomass and total biomass vs. precipitation, and the quotient of shoot biomass and total biomass vs. temperature, respectively.


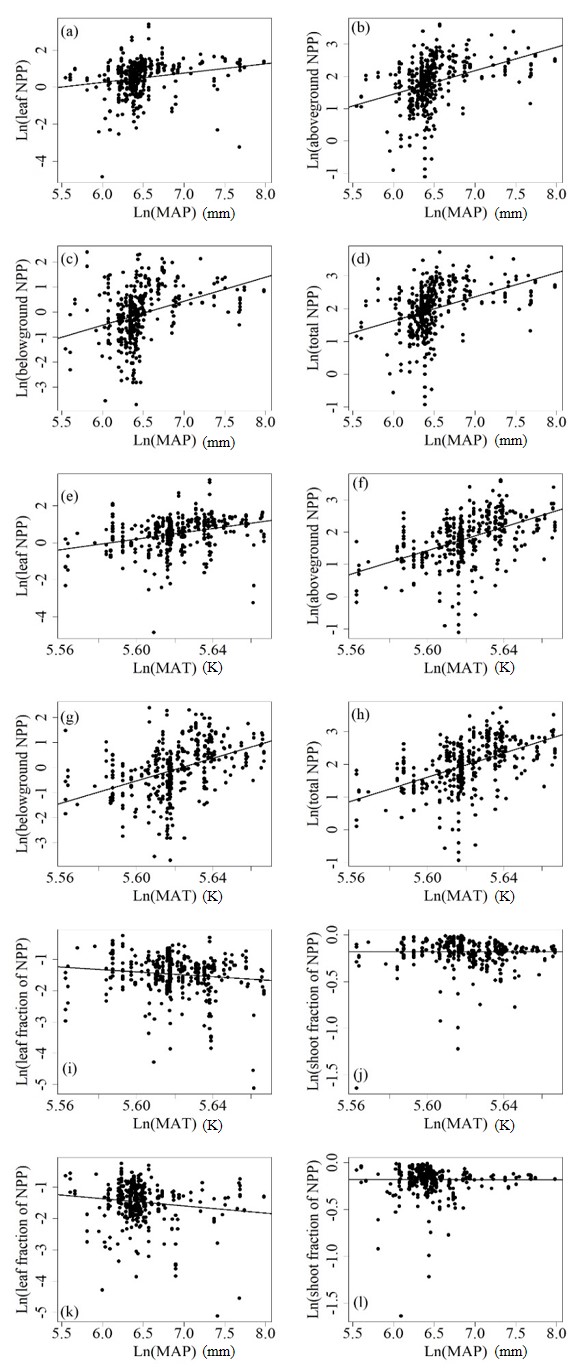


**Figure S3. Effect of climate on plant NPP (g m^−2^ yr^−1^) for different organ compartments. All variables are ln-transformed**. **(a-d)** Effects of MAP on leaf NPP, aboveground NPP, belowground NPP, and total NPP, respectively. **(e-h)** Effects of MAT on leaf NPP, aboveground NPP, belowground NPP, and total NPP, respectively. **(i, j)** Effects of MAT on leaf and shoot fractions of NPP, respectively. **(k, l)** Effects of MAP on leaf and shoot fractions of NPP, respectively.


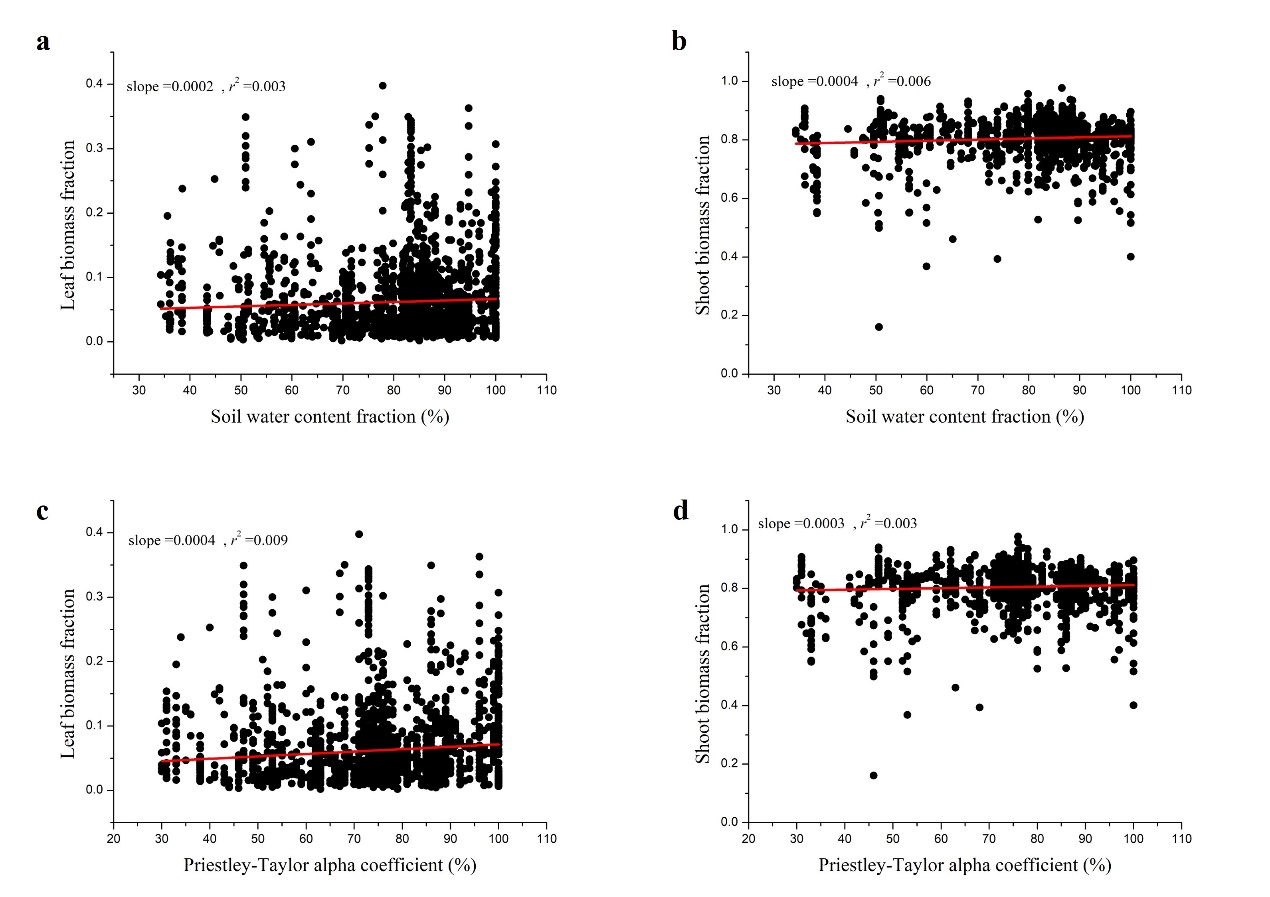


**Figure S4. Effect of soil water balance on biomass fractions. (a-b)** Effects of soil water content fraction on leaf and shoot biomass fractions, respectively. (**c-d**) Effects of the Priestley-Taylor alpha coefficient on leaf and shoot biomass fractions, respectively.

**
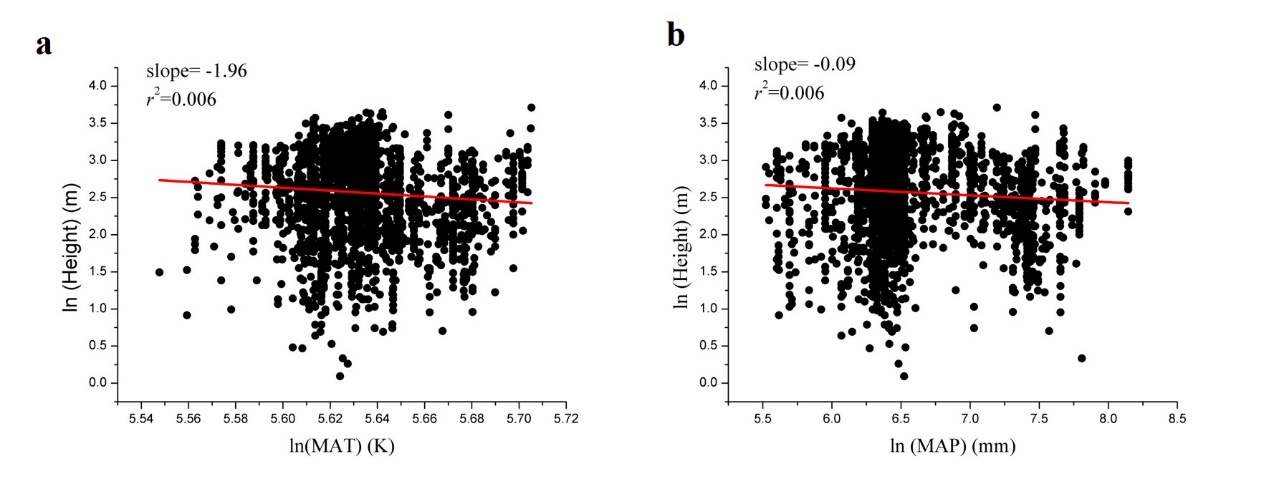
**

**Figure S5. Effect of temperature (a) and precipitation (b) on plant height.**


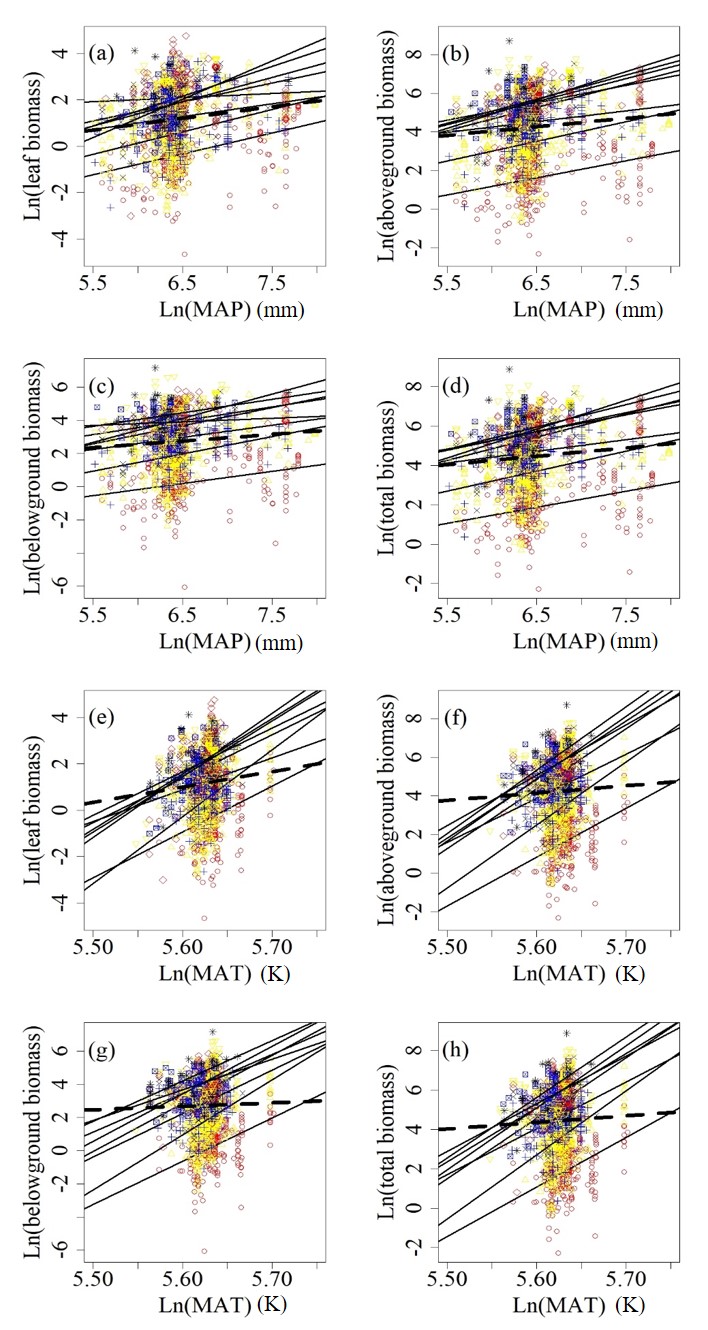


**Figure S6. Effect of climate on plant biomass for different organ compartments under different plant age classes. All variables are ln-transformed. (a-d)** Effect of MAP (mm) on leaf biomass (Kg), aboveground biomass (Kg), belowground biomass (Kg), and total biomass (Kg). **(e-h)** Effect of MAT (K) on leaf biomass (Kg), aboveground biomass (Kg), belowground biomass (Kg), and total biomass (Kg). Different colours and shapes of the scatter points in each subplot represent different age classes: 0-20 years (red circle), 21-40 years (yellow triangle), 41-60 years (blue plus sign), 61-80 years (black multiplication sign), 81-100 years (red diamond), 101-150 years (yellow triangle pointing down), 151-200 years (filled square), >200 years (black star). The dashed fitting lines represent the regression analysis with the total data set among the different age classes, while the solid lines are the regression analysis within the different age classes.

**Table S1.** V**alues of variance inflation factors (VIFs) for each covariate in each model, reflecting the relationships of leaf biomass fraction and biotic/abiotic factors.** Models with and without covariates of total biomass were compared. Strong co-linearity can be observed in all models with covariates of total biomass, as the VIFs are larger than 10 (three columns in left), while the VIFs for all models without covariates of total biomass are much smaller than 10 (three columns on the right).

| ***n*** | ***Model*** | ***Covariate*** | ***VIF*** | ***Model*** | ***Covariate*** | ***VIF*** |
| --- | --- | --- | --- | --- | --- | --- |
| 2347 | *H, N, M_T_* | *H* | 6.384 | *H, N* | *H* | 2.024 |
|  |  | *N* | 5.476 |  | *N* | 2.024 |
|  |  | *M_T_* | 14.332 |  |  |  |
| 2347 | *H, N, T, M_T_* | *H* | 6.482 | *H, N, T* | *H* | 2.024 |
|  |  | *N* | 5.633 |  | *N* | 2.038 |
|  |  | *T* | 1.036 |  | *T* | 1.013 |
|  |  | *M_T_* | 14.654 |  |  |  |
| 2347 | *H, N, P, M_T_* | *H* | 6.968 | *H, N, P* | *H* | 2.032 |
|  |  | *N* | 5.819 |  | *N* | 2.024 |
|  |  | *P* | 1.110 |  | *P* | 1.007 |
|  |  | *M_T_* | 15.812 |  |  |  |
| 2347 | *H, N, P, T, M_T_* | *H* | 7.056 | *H, N, P, T* | *H* | 2.040 |
|  |  | *N* | 5.819 |  | *N* | 2.054 |
|  |  | *P* | 2.124 |  | *P* | 1.951 |
|  |  | *T* | 1.981 |  | *T* | 1.964 |
|  |  | *M_T_* | 15.954 |  |  |  |

*H*, *N*, *M_T_*, *P*, and *T* represent plant height (m), plant density (trees/ha), plant total biomass (Kg), annual mean precipitation (MAP, mm) and annual mean temperature (MAT, K), respectively.

**Table S2.** **Candidate models for the relationship between leaf biomass fraction and influencing factors (including biotic and abiotic). Models are ranked according to the value of ΔAIC for discovering the model with the best AIC based on the principle that the model with the lowest ΔAIC is the model with the best AIC.**

| ***Rank*** | ***K*** | ***df*** | ***Candidate Model*** | ***AIC*** | ***Δ AIC*** | ***Ω*** |
| --- | --- | --- | --- | --- | --- | --- |
| 1 | 3 | 2343 | *H, N, T* | 4819.319 | 0.000 | 0.672 |
| 2 | 4 | 2342 | *H, N, P, T* | 4820.755 | 1.436 | 0.328 |
| 3 | 3 | 2343 | *H, N, P* | 4839.759 | 20.440 | 0.000 |
| 4 | 2 | 2344 | *H, T* | 4842.844 | 23.525 | 0.000 |
| 5 | 3 | 2343 | *H, P, T* | 4844.756 | 25.437 | 0.000 |
| 6 | 2 | 2344 | *H, N* | 4867.497 | 48.178 | 0.000 |
| 7 | 2 | 2344 | *H, P* | 4869.790 | 50.471 | 0.000 |
| 8 | 1 | 2345 | *H* | 4896.658 | 77.339 | 0.000 |
| 9 | 3 | 2343 | *N, P, T* | 5339.578 | 520.259 | 0.000 |
| 10 | 2 | 2344 | *N, T* | 5344.117 | 524.798 | 0.000 |
| 11 | 2 | 2344 | *N, P* | 5345.386 | 526.067 | 0.000 |
| 12 | 1 | 2345 | *N* | 5382.054 | 562.735 | 0.000 |
| 13 | 1 | 2345 | *T* | 6029.616 | 1210.297 | 0.000 |
| 14 | 2 | 2344 | *P, T* | 6030.027 | 1210.708 | 0.000 |
| 15 | 1 | 2345 | *P* | 6053.971 | 1234.652 | 0.000 |

*H*, *N*, *P*, and *T* represent the variables (contained in each candidate model) of plant height (m), plant density (trees/ha), annual mean precipitation (MAP, mm) and annual mean temperature (MAT, K), respectively; ***K*** is the number of estimated parameters in each candidate model; ***df*** represents the degrees of freedom; **AIC** is the Akaike information criterion; **Δ AIC** = **AIC** – min (**AIC**); ***ω*** is the rounded Akaike weight.

**Table S3. Summary of scaling relationships between two variables for different plant families.** *M_S,_ M_L_*, *M_T,_ H, N, P,* and *T,* represent plant aboveground biomass (Kg), plant leaf biomass (Kg), plant total biomass (Kg), plant height (m), plant density (trees/ha), annual mean precipitation (MAP, mm) and annual mean temperature (MAT, K), respectively. All variables are ln-transformed.

| ***Taxon*** | ***n*** | ***Y_1_*** | ***Y_2_*** | ***slope*** | ***95% CI*** | ***r^2^*** | ***P*** |
| --- | --- | --- | --- | --- | --- | --- | --- |
| Betulaceae | 184 | *M_L_*/*M_T_* | *H* | -0.460 | -0.615, -0.305 | 0.158 | **0.000** |
|  | 184 | *M_L_*/*M_T_* | *N* | 0.364 | 0.285, 0.444 | 0.311 | **0.000** |
|  | 184 | *M_L_*/*M_T_* | *P* | 0.244 | -0.187, 0.676 | 0.007 | 0.266 |
|  | 184 | *M_L_*/*M_T_* | *T* | -1.249 | -7.257, 4.759 | 0.001 | 0.682 |
|  | 68 | *M_S_*/*M_T_* | H | 0.038 | 0.001, 0.075 | 0.058 | **0.047** |
|  | 68 | *M_S_*/*M_T_* | N | -0.018 | -0.039, 0.002 | 0.045 | 0.083 |
|  | 68 | *M_S_*/*M_T_* | P | 0.100 | 0.032, 0.168 | 0.116 | **0.004** |
|  | 68 | *M_S_*/*M_T_* | T | 0.747 | -0.447, 1.941 | 0.023 | 0.216 |
| Cupressaceae | 24 | *M_L_*/*M_T_* | *H* | -0.541 | -1.012, -0.071 | 0.205 | **0.026** |
|  | 24 | *M_L_*/*M_T_* | *N* | 0.662 | 0.163, 1.161 | 0.256 | **0.012** |
|  | 24 | *M_L_*/*M_T_* | *P* | -0.065 | -0.51, 0.379 | 0.004 | 0.764 |
|  | 24 | *M_L_*/*M_T_* | *T* | 12.079 | -2.162, 26.321 | 0.123 | 0.092 |
|  | 14 | *M_S_*/*M_T_* | H | 0.012 | -0.071, 0.094 | 0.008 | 0.763 |
|  | 14 | *M_S_*/*M_T_* | N | 0.005 | -0.070, 0.081 | 0.002 | 0.879 |
|  | 14 | *M_S_*/*M_T_* | P | 0.031 | -0.019, 0.081 | 0.132 | 0.201 |
|  | 14 | *M_S_*/*M_T_* | T | 1.646 | 0.691, 2.600 | 0.541 | 0.003 |
| Dipterocarpaceae | 25 | *M_L_*/*M_T_* | *H* | -1.214 | -1.367, -1.060 | 0.921 | **0.000** |
|  | 25 | *M_L_*/*M_T_* | *N* | 0.263 | -0.162, 0.689 | 0.066 | 0.213 |
|  | 25 | *M_L_*/*M_T_* | *P* | 3.050 | -0.978, 7.077 | 0.096 | 0.131 |
|  | 25 | *M_L_*/*M_T_* | *T* | 568.987 | -347.144, 1485.118 | 0.067 | 0.212 |
|  | 12 | *M_S_*/*M_T_* | H | 0.052 | 0.009, 0.094 | 0.422 | **0.022** |
|  | 12 | *M_S_*/*M_T_* | N | -0.064 | -0.116, -0.011 | 0.420 | **0.023** |
|  | 12 | *M_S_*/*M_T_* | P | 0.295 | -0.683, 1.273 | 0.043 | 0.516 |
| Fagaceae | 262 | *M_L_*/*M_T_* | *H* | -0.953 | -1.08, -0.827 | 0.458 | **0.000** |
|  | 262 | *M_L_*/*M_T_* | *N* | 0.480 | 0.423, 0.536 | 0.517 | **0.000** |
|  | 262 | *M_L_*/*M_T_* | *P* | -0.161 | -0.332, 0.010 | 0.013 | 0.064 |
|  | 262 | *M_L_*/*M_T_* | *T* | 25.583 | 19.765, 31.401 | 0.224 | 0.000 |
|  | 146 | *M_S_*/*M_T_* | H | 0.236 | 0.191, 0.280 | 0.429 | **0.000** |
|  | 146 | *M_S_*/*M_T_* | N | -0.096 | -0.122, -0.071 | 0.278 | **0.000** |
|  | 146 | *M_S_*/*M_T_* | P | 0.085 | 0.022, 0.148 | 0.047 | **0.009** |
|  | 146 | *M_S_*/*M_T_* | T | -0.802 | -2.677, 1.073 | 0.005 | 0.399 |
| Lauraceae | 9 | *M_L_*/*M_T_* | *H* | -0.758 | -2.180, 0.664 | 0.185 | 0.248 |
|  | 9 | *M_L_*/*M_T_* | *N* | 0.835 | 0.130, 1.540 | 0.528 | **0.027** |
|  | 9 | *M_L_*/*M_T_* | *P* | 3.124 | -6.791, 13.039 | 0.073 | 0.481 |
|  | 9 | *M_L_*/*M_T_* | *T* | -53.383 | -207.601, 100.834 | 0.087 | 0.440 |
|  | 10 | *M_S_*/*M_T_* | H | 0.055 | -0.106, 0.217 | 0.072 | 0.452 |
|  | 10 | *M_S_*/*M_T_* | N | -0.021 | -0.127, 0.086 | 0.025 | 0.665 |
|  | 10 | *M_S_*/*M_T_* | P | -0.062 | -0.796, 0.671 | 0.005 | 0.849 |
|  | 10 | *M_S_*/*M_T_* | T | 0.993 | -6.957, 8.942 | 0.010 | 0.781 |
| Leguminosae | 24 | *M_L_/M_T_* | *H* | -1.256 | -1.585, -0.927 | 0.740 | **0.000** |
|  | 24 | *M_L_/M_T_* | *N* | 0.490 | 0.186, 0.794 | 0.337 | **0.003** |
|  | 24 | *M_L_/M_T_* | *P* | 1.241 | 0.511, 1.972 | 0.361 | **0.002** |
|  | 24 | *M_L_/M_T_* | *T* | 11.261 | -9.217, 31.740 | 0.056 | 0.266 |
|  | 16 | *M_S_*/*M_T_* | H | 0.035 | -0.001, 0.071 | 0.234 | 0.057 |
|  | 16 | *M_S_*/*M_T_* | N | -0.031 | -0.052, -0.009 | 0.405 | **0.008** |
|  | 16 | *M_S_*/*M_T_* | P | 0.003 | -0.044, 0.050 | 0.002 | 0.883 |
|  | 16 | *M_S_*/*M_T_* | T | 0.205 | -0.835, 1.244 | 0.013 | 0.679 |
| Myrtaceae | 16 | *M_L_*/*M_T_* | *H* | -1.269 | -2.122, -0.416 | 0.421 | **0.007** |
|  | 16 | *M_L_*/*M_T_* | *N* | 0.928 | 0.154, 1.702 | 0.321 | **0.022** |
|  | 16 | *M_L_*/*M_T_* | *P* | -0.621 | -1.692, 0.449 | 0.100 | 0.233 |
|  | 16 | *M_L_*/*M_T_* | *T* | -13.992 | -49.558, 21.573 | 0.048 | 0.413 |
|  | 18 | *M_S_*/*M_T_* | H | 0.054 | -0.043, 0.150 | 0.080 | 0.256 |
|  | 18 | *M_S_*/*M_T_* | N | 0.005 | -0.068, 0.078 | 0.001 | 0.882 |
|  | 18 | *M_S_*/*M_T_* | P | -0.079 | -0.177, 0.018 | 0.157 | 0.104 |
|  | 18 | *M_S_*/*M_T_* | T | 1.996 | -1.432, 5.425 | 0.087 | 0.235 |
| Pinaceae | 1473 | *M_L_*/*M_T_* | *H* | -0.792 | -0.841, -0.744 | 0.412 | **0.000** |
|  | 1473 | *M_L_*/*M_T_* | *N* | 0.388 | 0.356, 0.419 | 0.285 | **0.000** |
|  | 1473 | *M_L_*/*M_T_* | *P* | 0.184 | 0.076, 0.291 | 0.008 | **0.001** |
|  | 1473 | *M_L_*/*M_T_* | *T* | 5.988 | 3.891, 8.085 | 0.021 | 0.000 |
|  | 800 | *M_S_*/*M_T_* | H | 0.011 | 0.002, 0.02 | 0.007 | **0.017** |
|  | 800 | *M_S_*/*M_T_* | N | -0.002 | -0.008, 0.003 | 0.001 | 0.360 |
|  | 800 | *M_S_*/*M_T_* | P | 0.029 | 0.014, 0.044 | 0.017 | **0.000** |
|  | 800 | *M_S_*/*M_T_* | T | 0.603 | 0.335, 0.871 | 0.024 | 0.000 |
| Salicaceae | *58* | *M_L_*/*M_T_* | *H* | -1.048 | -1.308, -0.788 | 0.538 | **0.000** |
|  | *58* | *M_L_*/*M_T_* | *N* | 0.216 | 0.064, 0.368 | 0.127 | **0.006** |
|  | *58* | *M_L_*/*M_T_* | *P* | -0.720 | -1.583, 0.144 | 0.047 | 0.101 |
|  | *58* | *M_L_*/*M_T_* | *T* | 3.347 | -4.572, 11.265 | 0.013 | 0.401 |
|  | 34 | *M_S_*/*M_T_* | H | 0.102 | 0.035, 0.170 | 0.230 | **0.004** |
|  | 34 | *M_S_*/*M_T_* | N | -0.013 | -0.045, 0.019 | 0.021 | 0.410 |
|  | 34 | *M_S_*/*M_T_* | P | 0.284 | 0.132, 0.437 | 0.311 | **0.001** |
|  | 34 | *M_S_*/*M_T_* | T | -1.037 | -2.264, 0.191 | 0.085 | 0.095 |
| Taxodiaceae | 188 | *M_L_*/*M_T_* | *H* | -1.114 | -1.252, -0.975 | 0.574 | **0.000** |
|  | 188 | *M_L_*/*M_T_* | *N* | 0.471 | 0.360, 0.582 | 0.275 | **0.000** |
|  | 188 | *M_L_*/*M_T_* | *P* | 1.243 | 0.784, 1.702 | 0.133 | **0.000** |
|  | 188 | *M_L_*/*M_T_* | *T* | -19.798 | -26.975, -12.622 | 0.137 | 0.000 |
|  | 151 | *M_S_*/*M_T_* | H | 0.053 | 0.034, 0.072 | 0.165 | **0.000** |
|  | 151 | *M_S_*/*M_T_* | N | -0.010 | -0.025, 0.006 | 0.010 | 0.217 |
|  | 151 | *M_S_*/*M_T_* | P | 0.043 | -0.011, 0.096 | 0.016 | 0.117 |
|  | 151 | *M_S_*/*M_T_* | T | -0.843 | -1.631, -0.054 | 0.029 | 0.036 |
| Total forest | 2347 | *M_L_*/*M_T_* | *H* | -0.944 | -0.992, -0.898 | 0.401 | **0.000** |
|  | 2347 | *M_L_*/*M_T_* | *N* | 0.432 | 0.403, 0.462 | 0.263 | **0.000** |
|  | 2347 | *M_L_*/*M_T_* | *P* | 0.234 | 0.166, 0.304 | 0.019 | **0.000** |
|  | 2347 | *M_L_*/*M_T_* | *T* | 6.014 | 4.591, 7.437 | 0.028 | **0.000** |
|  | 1349 | *M_S_*/*M_T_* | H | 0.038 | 0.028, 0.047 | 0.044 | **0.000** |
|  | 1349 | *M_S_*/*M_T_* | N | -0.020 | -0.026, -0.015 | 0.038 | **0.000** |
|  | 1349 | *M_S_*/*M_T_* | P | 0.002 | -0.008, 0.012 | 0.000 | 0.713 |
|  | 1349 | *M_S_*/*M_T_* | T | -0.103 | -0.298, 0.092 | 0.000 | 0.300 |

**Table S4. Partial regression relationships between leaf biomass fraction and four influencing factors for different families as well as total pooled data.** *H, N, P,* and *T* represent plant height (m), plant density (trees/ha), annual mean precipitation (MAP, mm) and annual mean temperature (MAT, K), respectively. All variables are ln-transformed.

| ***Family*** | ***n*** | ***Variable*** | ***Coefficient*** | ***Std. Error*** | ***t value*** | ***P*** | ***Partial r^2^*** |
| --- | --- | --- | --- | --- | --- | --- | --- |
| *Betulaceae* |  | intercept | 28.329 | 16.812 | 1.685 | 0.094 |  |
|  | 184 | *H* | -0.031 | 0.101 | -0.312 | 0.755 | 0 |
|  | 184 | *N* | 0.364 | 0.056 | 6.519 | 0 | 0.155 |
|  | 184 | *P* | 0.686 | 0.226 | 3.038 | 0.003 | 0.034 |
|  | 184 | *T* | -6.956 | 3.149 | -2.209 | 0.028 | 0.018 |
| *Cupressaceae* |  | intercept | -46.047 | 38.705 | -1.19 | 0.249 |  |
|  | 24 | *H* | -0.374 | 0.437 | -0.856 | 0.403 | 0.025 |
|  | 24 | *N* | 0.392 | 0.352 | 1.112 | 0.28 | 0.042 |
|  | 24 | *P* | 0.115 | 0.309 | 0.37 | 0.715 | 0.005 |
|  | 24 | *T* | 7.172 | 6.812 | 1.053 | 0.306 | 0.037 |
| *Dipterocarpaceae* |  | intercept | 6496.847 | 2980.043 | 2.18 | 0.041 |  |
|  | 25 | *H* | -1.467 | 0.148 | -9.922 | 0 | 0.3 |
|  | 25 | *N* | -0.323 | 0.182 | -1.772 | 0.092 | 0.01 |
|  | 25 | *P* | 1.31 | 1.532 | 0.855 | 0.403 | 0.002 |
|  | 25 | *T* | -1141.422 | 524.029 | -2.178 | 0.042 | 0.014 |
| *Fagaceae* |  | intercept | -99.556 | 11.378 | -8.75 | 0 |  |
|  | 262 | *H* | -0.56 | 0.086 | -6.515 | 0 | 0.056 |
|  | 262 | *N* | 0.214 | 0.041 | 5.196 | 0 | 0.035 |
|  | 262 | *P* | -0.174 | 0.056 | -3.101 | 0.002 | 0.013 |
|  | 262 | *T* | 17.144 | 2.024 | 8.472 | 0 | 0.094 |
| *Lauraceae* |  | intercept | 393.521 | 382.463 | 1.029 | 0.362 |  |
|  | 9 | *H* | 0.457 | 0.996 | 0.459 | 0.67 | 0.019 |
|  | 9 | *N* | 0.925 | 0.416 | 2.227 | 0.09 | 0.446 |
|  | 9 | *P* | 1.935 | 5.013 | 0.386 | 0.719 | 0.013 |
|  | 9 | *T* | -73.823 | 69.736 | -1.059 | 0.349 | 0.101 |
| *Leguminosae* |  | intercept | -117.753 | 32.845 | -3.585 | 0.002 |  |
|  | 24 | *H* | -1.405 | 0.18 | -7.829 | 0 | 0.299 |
|  | 24 | *N* | -0.009 | 0.089 | -0.096 | 0.924 | 0 |
|  | 24 | *P* | -0.065 | 0.275 | -0.237 | 0.815 | 0 |
|  | 24 | *T* | 20.951 | 6.099 | 3.435 | 0.003 | 0.058 |
| *Myrtaceae* |  | intercept | 232.388 | 193.837 | 1.199 | 0.256 |  |
|  | 16 | *H* | -0.946 | 0.585 | -1.617 | 0.134 | 0.115 |
|  | 16 | *N* | -0.459 | 0.979 | -0.469 | 0.648 | 0.01 |
|  | 16 | *P* | -1.407 | 1.382 | -1.018 | 0.331 | 0.046 |
|  | 16 | *T* | -38.685 | 31.569 | -1.225 | 0.246 | 0.066 |
| *Pinaceae* |  | intercept | -27.759 | 5.697 | -4.873 | 0 |  |
|  | 1473 | *H* | -0.67 | 0.035 | -18.924 | 0 | 0.137 |
|  | 1473 | *N* | 0.096 | 0.021 | 4.601 | 0 | 0.008 |
|  | 1473 | *P* | 0.05 | 0.054 | 0.932 | 0.351 | 0 |
|  | 1473 | *T* | 4.52 | 1.054 | 4.29 | 0 | 0.007 |
| *Salicaceae* |  | intercept | -31.515 | 13.968 | -2.256 | 0.028 |  |
|  | 58 | *H* | -1.977 | 0.199 | -9.936 | 0 | 0.532 |
|  | 58 | *N* | -0.466 | 0.083 | -5.636 | 0 | 0.171 |
|  | 58 | *P* | 0.738 | 0.299 | 2.47 | 0.017 | 0.033 |
|  | 58 | *T* | 5.72 | 2.395 | 2.388 | 0.021 | 0.031 |
| *Taxodiaceae* |  | intercept | 106.42 | 18.6 | 5.721 | 0 |  |
|  | 188 | *H* | -1.186 | 0.07 | -16.83 | 0 | 0.364 |
|  | 188 | *N* | -0.027 | 0.043 | -0.627 | 0.531 | 0.001 |
|  | 188 | *P* | 0.341 | 0.196 | 1.746 | 0.083 | 0.004 |
|  | 188 | *T* | -19.109 | 3.071 | -6.222 | 0 | 0.05 |
| *Total forest* |  | intercept | -21.779 | 4.252 | -5.123 | 0 |  |
|  | 2347 | *H* | -0.808 | 0.034 | -24.097 | 0 | 0.144 |
|  | 2347 | *N* | 0.098 | 0.019 | 5.138 | 0 | 0.007 |
|  | 2347 | *P* | 0.031 | 0.038 | 0.823 | 0.411 | 0 |
|  | 2347 | *T* | 3.509 | 0.788 | 4.452 | 0 | 0.005 |

**Table S5. Partial regression relationships between shoot biomass fraction and four influencing factors for different families as well as total pooled data.** *H, N, P,* and *T* represent plant height (m), plant density (trees/ha), annual mean precipitation (MAP, mm) and annual mean temperature (MAT, K), respectively. All variables are ln-transformed.

| ***Family*** | ***n*** | ***Variable*** | ***Coefficient*** | ***Std. Error*** | ***t value*** | ***P*** | ***Partial r^2^*** |
| --- | --- | --- | --- | --- | --- | --- | --- |
| *Betulaceae* |  | intercept | 6.705 | 4.712 | 1.423 | 0.160 |  |
|  | 68 | *H* | 0.016 | 0.027 | 0.586 | 0.560 | 0.004 |
|  | 68 | *N* | -0.010 | 0.015 | -0.630 | 0.531 | 0.005 |
|  | 68 | *P* | 0.156 | 0.053 | 2.942 | 0.005 | 0.112 |
|  | 68 | *T* | -1.404 | 0.881 | -1.592 | 0.116 | 0.033 |
| *Cupressaceae* |  | intercept | -14.332 | 5.543 | -2.585 | 0.029 |  |
|  | 14 | *H* | 0.113 | 0.103 | 1.095 | 0.302 | 0.051 |
|  | 14 | *N* | 0.029 | 0.049 | 0.587 | 0.571 | 0.015 |
|  | 14 | *P* | -0.069 | 0.077 | -0.891 | 0.396 | 0.034 |
|  | 14 | *T* | 2.495 | 0.984 | 2.535 | 0.032 | 0.275 |
| *Fagaceae* |  | intercept | 4.568 | 3.938 | 1.160 | 0.248 |  |
|  | 146 | *H* | 0.288 | 0.039 | 7.391 | 0.000 | 0.197 |
|  | 146 | *N* | 0.033 | 0.020 | 1.629 | 0.105 | 0.010 |
|  | 146 | *P* | 0.100 | 0.025 | 4.012 | 0.000 | 0.058 |
|  | 146 | *T* | -1.154 | 0.711 | -1.624 | 0.107 | 0.010 |
| *Lauraceae* |  | intercept | -0.713 | 49.023 | -0.015 | 0.989 |  |
|  | 10 | *H* | 0.050 | 0.170 | 0.296 | 0.779 | 0.016 |
|  | 10 | *N* | -0.010 | 0.071 | -0.136 | 0.897 | 0.003 |
|  | 10 | *P* | 0.020 | 0.772 | 0.026 | 0.980 | 0.000 |
|  | 10 | *T* | 0.048 | 9.562 | 0.005 | 0.996 | 0.000 |
| *Leguminosae* |  | intercept | 0.334 | 4.690 | 0.071 | 0.945 |  |
|  | 16 | *H* | 0.000 | 0.029 | -0.015 | 0.988 | 0.000 |
|  | 16 | *N* | -0.031 | 0.018 | -1.700 | 0.117 | 0.156 |
|  | 16 | *P* | 0.000 | 0.041 | -0.012 | 0.991 | 0.000 |
|  | 16 | *T* | -0.054 | 0.875 | -0.062 | 0.952 | 0.000 |
| *Myrtaceae* |  | intercept | 46.068 | 15.866 | 2.904 | 0.012 |  |
|  | 18 | *H* | 0.120 | 0.049 | 2.462 | 0.029 | 0.154 |
|  | 18 | *N* | -0.180 | 0.077 | -2.331 | 0.037 | 0.138 |
|  | 18 | *P* | -0.437 | 0.113 | -3.861 | 0.002 | 0.378 |
|  | 18 | *T* | -7.427 | 2.582 | -2.876 | 0.013 | 0.209 |
| *Pinaceae* |  | intercept | -3.049 | 0.969 | -3.146 | 0.002 |  |
|  | 800 | *H* | 0.019 | 0.006 | 2.940 | 0.003 | 0.010 |
|  | 800 | *N* | 0.005 | 0.004 | 1.328 | 0.185 | 0.002 |
|  | 800 | *P* | 0.014 | 0.010 | 1.353 | 0.177 | 0.002 |
|  | 800 | *T* | 0.474 | 0.180 | 2.630 | 0.009 | 0.008 |
| *Salicaceae* |  | intercept | -0.066 | 3.400 | -0.019 | 0.985 |  |
|  | 34 | *H* | 0.076 | 0.052 | 1.465 | 0.154 | 0.042 |
|  | 34 | *N* | 0.001 | 0.022 | 0.030 | 0.976 | 0.000 |
|  | 34 | *P* | 0.213 | 0.102 | 2.091 | 0.045 | 0.086 |
|  | 34 | *T* | -0.307 | 0.560 | -0.547 | 0.589 | 0.006 |
| *Taxodiaceae* |  | intercept | 2.714 | 3.632 | 0.747 | 0.456 |  |
|  | 151 | *H* | 0.074 | 0.012 | 6.018 | 0.000 | 0.191 |
|  | 151 | *N* | 0.025 | 0.009 | 2.742 | 0.007 | 0.040 |
|  | 151 | *P* | 0.010 | 0.041 | 0.252 | 0.802 | 0.000 |
|  | 151 | *T* | -0.595 | 0.597 | -0.996 | 0.321 | 0.005 |
| *Total forest* |  | intercept | 0.819 | 0.795 | 1.030 | 0.303 |  |
|  | 1349 | *H* | 0.027 | 0.007 | 4.017 | 0.000 | 0.011 |
|  | 1349 | *N* | -0.009 | 0.004 | -2.455 | 0.014 | 0.004 |
|  | 1349 | *P* | 0.014 | 0.008 | 1.732 | 0.084 | 0.002 |
|  | 1349 | *T* | -0.200 | 0.148 | -1.351 | 0.177 | 0.001 |

**Table S6. Summary of analysis results of multiple regression analysis between leaf biomass fraction and four influencing factors for each of the three forest data sets.**

| ***Data resource*** | ***n*** | ***Variable*** | ***Coefficients*** | ***Std. error*** | ***t value*** | ***P value*** | ***Partial r^2^*** |
| --- | --- | --- | --- | --- | --- | --- | --- |
| Cannell, 1982 | 185 | intercept | -20.074 | 13.067 | -1.536 | 0.126 |  |
|  |  | *H* | -1.356 | 0.181 | -7.481 | 0.000 | 0.178 |
|  |  | *N* | 0.078 | 0.075 | 1.048 | 0.296 | 0.004 |
|  |  | *P* | -0.064 | 0.122 | -0.525 | 0.600 | 0.001 |
|  |  | *T* | 3.595 | 2.426 | 1.482 | 0.140 | 0.007 |
| Luo *et al.*, 2014 | 313 | intercept | 18.370 | 11.956 | 1.537 | 0.125 |  |
|  |  | *H* | -1.106 | 0.068 | -16.183 | 0.000 | 0.342 |
|  |  | *N* | 0.125 | 0.063 | 1.976 | 0.049 | 0.005 |
|  |  | *P* | 0.053 | 0.106 | 0.499 | 0.618 | 0.000 |
|  |  | *T* | -3.492 | 2.212 | -1.579 | 0.115 | 0.003 |
| Usoltsev, 2001 | 1849 | intercept | -13.665 | 5.563 | -2.456 | 0.014 |  |
|  |  | *H* | -0.639 | 0.039 | -16.455 | 0.000 | 0.093 |
|  |  | *N* | 0.140 | 0.021 | 6.561 | 0.000 | 0.015 |
|  |  | *P* | 0.052 | 0.047 | 1.105 | 0.269 | 0.000 |
|  |  | *T* | 1.904 | 1.026 | 1.856 | 0.064 | 0.001 |

*P*, *N*, *T*, and *H* represent precipitation (mm), plant density (trees/ha), temperature (K) and plant height (m), respectively.

**Table S7. Summary analysis results of the regression relationships between plant NPP/biomass traits vs. climate (MAP and MAT).**

| Regression relationship | *n* | slope | *r*^2^ | 95% CI | *P* |
| --- | --- | --- | --- | --- | --- |
| Leaf NPP vs. MAP | 418 | 0.50 | 0.04 | 0.275, 0.725 | 0.00 |
| Aboveground NPP vs. MAP | 418 | 0.73 | 0.14 | 0.556, 0.899 | 0.00 |
| Belowground NPP vs. MAP | 418 | 0.95 | 0.12 | 0.704, 1.202 | 0.00 |
| Total NPP vs. MAP | 418 | 0.73 | 0.14 | 0.557, 0.900 | 0.00 |
| Leaf NPP vs. MAT | 418 | 14.34 | 0.10 | 10.193, 18.484 | 0.00 |
| Aboveground NPP vs. MAT | 418 | 18.25 | 0.25 | 15.196, 21.298 | 0.00 |
| Belowground NPP vs. MAT | 418 | 22.57 | 0.19 | 18.029, 27.111 | 0.00 |
| Total NPP vs. MAT | 418 | 18.25 | 0.25 | 15.201, 21.293 | 0.00 |
| Leaf NPP fraction vs. MAT | 418 | -3.91 | 0.02 | -6.895, -0.923 | 0.01 |
| Shoot NPP fraction vs. MAT | 418 | 0.00 | 0.00 | -0.725, 0.724 | 0.99 |
| Leaf NPP fraction vs. MAP | 418 | -0.23 | 0.02 | -0.386, -0.072 | 0.01 |
| Shoot NPP fraction vs. MAP | 418 | 0.00 | 0.00 | -0.039, 0.037 | 0.95 |
| Leaf biomass vs. MAP | 2347 | 0.27 | 0.01 | 0.172, 0.368 | 0.00 |
| Total biomass vs. MAP | 2347 | 0.04 | 0.00 | -0.093, 0.163 | 0.59 |
| Aboveground biomass vs. MAP | 1349 | -0.05 | 0.00 | -0.208, 0.103 | 0.50 |
| Belowground biomass vs. MAP | 1349 | -0.03 | 0.00 | -0.186, 0.12 | 0.67 |
| Leaf biomass vs. MAT | 2347 | 1.95 | 0.00 | -0.082, 3.991 | 0.06 |
| Total biomass vs. MAT | 2347 | -4.06 | 0.00 | -6.691, -1.427 | 0.00 |
| Aboveground biomass vs. MAT | 1349 | -4.96 | 0.01 | -7.88, -2.031 | 0.00 |
| Belowground biomass vs. MAT | 1349 | -4.51 | 0.01 | -7.385, -1.634 | 0.00 |

**Table S8. Summary analysis results of the regression relationships between plant biomass traits vs. climate (MAP and MAT) for different plant age classes.**

| Regression relationship | Age class (year) | *n* | slope | *r*^2^ | 95% CI | *P* |
| --- | --- | --- | --- | --- | --- | --- |
| Aboveground biomass vs. MAT | 0-20 | 238 | 25.3 | 0.17 | 18.252, 32.354 | 0.00 |
| Aboveground biomass vs. MAT | 21-40 | 433 | 32.61 | 0.19 | 26.186, 39.037 | 0.00 |
| Aboveground biomass vs. MAT | 41-60 | 300 | 22.84 | 0.11 | 15.384, 30.297 | 0.00 |
| Aboveground biomass vs. MAT | 61-80 | 208 | 31.08 | 0.18 | 21.978, 40.181 | 0.00 |
| Aboveground biomass vs. MAT | 81-100 | 217 | 30.29 | 0.20 | 22.153, 38.432 | 0.00 |
| Aboveground biomass vs. MAT | 101-150 | 226 | 32.23 | 0.25 | 24.937, 39.531 | 0.00 |
| Aboveground biomass vs. MAT | 151-200 | 78 | 26.11 | 0.19 | 13.837, 38.374 | 0.00 |
| Aboveground biomass vs. MAT | >200 | 35 | 32.72 | 0.40 | 18.585, 46.852 | 0.00 |
| Belowground biomass vs. MAT | 0-20 | 238 | 26 | 0.17 | 18.596, 33.405 | 0.00 |
| Belowground biomass vs. MAT | 21-40 | 433 | 33.09 | 0.19 | 26.659, 39.519 | 0.00 |
| Belowground biomass vs. MAT | 41-60 | 300 | 26.23 | 0.14 | 18.944, 33.526 | 0.00 |
| Belowground biomass vs. MAT | 61-80 | 208 | 28.86 | 0.15 | 19.471, 38.24 | 0.00 |
| Belowground biomass vs. MAT | 81-100 | 217 | 23.44 | 0.14 | 15.717, 31.16 | 0.00 |
| Belowground biomass vs. MAT | 101-150 | 226 | 28.67 | 0.23 | 21.744, 35.591 | 0.00 |
| Belowground biomass vs. MAT | 151-200 | 78 | 18.43 | 0.13 | 7.502, 29.367 | 0.00 |
| Belowground biomass vs. MAT | >200 | 35 | 24.29 | 0.35 | 12.583, 36 | 0.00 |
| Leaf biomass vs. MAT | 0-20 | 238 | 19.79 | 0.11 | 12.547, 27.028 | 0.00 |
| Leaf biomass vs. MAT | 21-40 | 433 | 28.91 | 0.18 | 23.102, 34.716 | 0.00 |
| Leaf biomass vs. MAT | 41-60 | 300 | 13.74 | 0.04 | 6.264, 21.225 | 0.00 |
| Leaf biomass vs. MAT | 61-80 | 208 | 19 | 0.08 | 9.93, 28.07 | 0.00 |
| Leaf biomass vs. MAT | 81-100 | 217 | 26.76 | 0.15 | 18.179, 35.339 | 0.00 |
| Leaf biomass vs. MAT | 101-150 | 226 | 23.9 | 0.18 | 17.205, 30.593 | 0.00 |
| Leaf biomass vs. MAT | 151-200 | 78 | 23.95 | 0.20 | 12.994, 34.907 | 0.00 |
| Leaf biomass vs. MAT | >200 | 35 | 18.85 | 0.20 | 5.438, 32.254 | 0.01 |
| Total biomass vs. MAT | 0-20 | 238 | 25.21 | 0.18 | 18.365, 32.049 | 0.00 |
| Total biomass vs. MAT | 21-40 | 433 | 32.53 | 0.19 | 26.198, 38.87 | 0.00 |
| Total biomass vs. MAT | 41-60 | 300 | 23.54 | 0.12 | 16.198, 30.886 | 0.00 |
| Total biomass vs. MAT | 61-80 | 208 | 30.8 | 0.18 | 21.747, 39.86 | 0.00 |
| Total biomass vs. MAT | 81-100 | 217 | 27.5 | 0.18 | 19.674, 35.336 | 0.00 |
| Total biomass vs. MAT | 101-150 | 226 | 31.54 | 0.25 | 24.393, 38.688 | 0.00 |
| Total biomass vs. MAT | 151-200 | 78 | 24.19 | 0.18 | 12.452, 35.931 | 0.00 |
| Total biomass vs. MAT | >200 | 35 | 30.77 | 0.40 | 17.439, 44.093 | 0.00 |
| Aboveground biomass vs. MAP | 0-20 | 238 | 0.88 | 0.13 | 0.589, 1.175 | 0.00 |
| Aboveground biomass vs. MAP | 21-40 | 433 | 1.02 | 0.10 | 0.733, 1.3 | 0.00 |
| Aboveground biomass vs. MAP | 41-60 | 300 | 0.55 | 0.03 | 0.218, 0.887 | 0.00 |
| Aboveground biomass vs. MAP | 61-80 | 208 | 1.2 | 0.09 | 0.678, 1.725 | 0.00 |
| Aboveground biomass vs. MAP | 81-100 | 217 | 0.9 | 0.13 | 0.585, 1.215 | 0.00 |
| Aboveground biomass vs. MAP | 101-150 | 226 | 1.47 | 0.13 | 0.969, 1.981 | 0.00 |
| Aboveground biomass vs. MAP | 151-200 | 78 | 1.19 | 0.07 | 0.191, 2.192 | 0.02 |
| Aboveground biomass vs. MAP | >200 | 35 | 1.25 | 0.08 | -0.248, 2.755 | 0.10 |
| Belowground biomass vs. MAP | 0-20 | 238 | 0.74 | 0.08 | 0.421, 1.05 | 0.00 |
| Belowground biomass vs. MAP | 21-40 | 433 | 1.03 | 0.11 | 0.747, 1.315 | 0.00 |
| Belowground biomass vs. MAP | 41-60 | 300 | 0.73 | 0.06 | 0.4, 1.059 | 0.00 |
| Belowground biomass vs. MAP | 61-80 | 208 | 1.1 | 0.07 | 0.561, 1.632 | 0.00 |
| Belowground biomass vs. MAP | 81-100 | 217 | 0.83 | 0.13 | 0.538, 1.115 | 0.00 |
| Belowground biomass vs. MAP | 101-150 | 226 | 1.45 | 0.14 | 0.984, 1.922 | 0.00 |
| Belowground biomass vs. MAP | 151-200 | 78 | 0.22 | 0.00 | -0.667, 1.111 | 0.62 |
| Belowground biomass vs. MAP | >200 | 35 | 0.82 | 0.05 | -0.394, 2.028 | 0.18 |
| Leaf biomass vs. MAP | 0-20 | 238 | 0.9 | 0.14 | 0.609, 1.185 | 0.00 |
| Leaf biomass vs. MAP | 21-40 | 433 | 0.97 | 0.11 | 0.712, 1.219 | 0.00 |
| Leaf biomass vs. MAP | 41-60 | 300 | 0.55 | 0.04 | 0.228, 0.875 | 0.00 |
| Leaf biomass vs. MAP | 61-80 | 208 | 0.82 | 0.05 | 0.319, 1.325 | 0.00 |
| Leaf biomass vs. MAP | 81-100 | 217 | 0.18 | 0.00 | -0.164, 0.524 | 0.30 |
| Leaf biomass vs. MAP | 101-150 | 226 | 1.29 | 0.13 | 0.846, 1.733 | 0.00 |
| Leaf biomass vs. MAP | 151-200 | 78 | 1.65 | 0.16 | 0.801, 2.502 | 0.00 |
| Leaf biomass vs. MAP | >200 | 35 | 1.04 | 0.08 | -0.185, 2.273 | 0.09 |
| Total biomass vs. MAP | 0-20 | 238 | 0.82 | 0.12 | 0.534, 1.109 | 0.00 |
| Total biomass vs. MAP | 21-40 | 433 | 1.01 | 0.10 | 0.731, 1.29 | 0.00 |
| Total biomass vs. MAP | 41-60 | 300 | 0.58 | 0.04 | 0.252, 0.914 | 0.00 |
| Total biomass vs. MAP | 61-80 | 208 | 1.18 | 0.09 | 0.663, 1.706 | 0.00 |
| Total biomass vs. MAP | 81-100 | 217 | 0.86 | 0.13 | 0.562, 1.161 | 0.00 |
| Total biomass vs. MAP | 101-150 | 226 | 1.47 | 0.13 | 0.972, 1.96 | 0.00 |
| Total biomass vs. MAP | 151-200 | 78 | 0.95 | 0.05 | -0.01, 1.913 | 0.05 |
| Total biomass vs. MAP | >200 | 35 | 1.13 | 0.07 | -0.284, 2.554 | 0.11 |

**Table S9 Effects of soil nutrients (carbon and nitrogen) on plant leaf and shoot biomass fractions.**

| Regression relationship | soil depth (cm) | slope | *r*^2^ | 95% CI | *P* |
| --- | --- | --- | --- | --- | --- |
| leaf biomass fraction vs. organic carbon | 0-20 | 0.00 | 0.01 | 0.000, 0.000 | 0.00 |
| leaf biomass fraction vs. organic carbon | 20-40 | 0.00 | 0.00 | 0.000, 0.000 | 0.01 |
| leaf biomass fraction vs. organic carbon | 40-60 | 0.00 | 0.00 | 0.000, 0.000 | 0.03 |
| leaf biomass fraction vs. organic carbon | 60-80 | 0.00 | 0.00 | 0.000, 0.000 | 0.01 |
| leaf biomass fraction vs. organic carbon | 80-100 | 0.00 | 0.00 | 0.000, 0.000 | 0.02 |
| leaf biomass fraction vs. organic carbon | 100-150 | 0.00 | 0.00 | 0.000, 0.000 | 0.70 |
| leaf biomass fraction vs. organic carbon | 150-200 | 0.00 | 0.00 | 0.000, 0.000 | 0.82 |
| leaf biomass fraction vs. total nitrogen | 0-20 | 0.00 | 0.01 | -0.003, -0.001 | 0.00 |
| leaf biomass fraction vs. total nitrogen | 20-40 | 0.00 | 0.00 | -0.003, -0.001 | 0.00 |
| leaf biomass fraction vs. total nitrogen | 40-60 | 0.00 | 0.00 | -0.003, -0.001 | 0.00 |
| leaf biomass fraction vs. total nitrogen | 60-80 | 0.00 | 0.00 | -0.003, -0.001 | 0.00 |
| leaf biomass fraction vs. total nitrogen | 80-100 | 0.00 | 0.00 | -0.003, 0 | 0.02 |
| leaf biomass fraction vs. total nitrogen | 100-150 | 0.00 | 0.00 | -0.002, 0 | 0.11 |
| leaf biomass fraction vs. total nitrogen | 150-200 | 0.00 | 0.00 | -0.002, 0 | 0.13 |
| shoot biomass fraction vs. organic carbon | 0-20 | 0.00 | 0.00 | 0.000, 0.000 | 0.89 |
| shoot biomass fraction vs. organic carbon | 20-40 | 0.00 | 0.00 | 0.000, 0.000 | 0.60 |
| shoot biomass fraction vs. organic carbon | 40-60 | 0.00 | 0.00 | 0.000, 0.000 | 0.63 |
| shoot biomass fraction vs. organic carbon | 60-80 | 0.00 | 0.00 | 0.000, 0.000 | 0.62 |
| shoot biomass fraction vs. organic carbon | 80-100 | 0.00 | 0.00 | 0.000, 0.000 | 0.51 |
| shoot biomass fraction vs. organic carbon | 100-150 | 0.00 | 0.00 | 0.000, 0.000 | 0.54 |
| shoot biomass fraction vs. organic carbon | 150-200 | 0.00 | 0.00 | 0.000, 0.000 | 0.58 |
| shoot biomass fraction vs. total nitrogen | 0-20 | 0.00 | 0.00 | -0.002, 0.001 | 0.53 |
| shoot biomass fraction vs. total nitrogen | 20-40 | 0.00 | 0.00 | -0.002, 0.002 | 0.85 |
| shoot biomass fraction vs. total nitrogen | 40-60 | 0.00 | 0.00 | -0.002, 0.002 | 0.90 |
| shoot biomass fraction vs. total nitrogen | 60-80 | 0.00 | 0.00 | -0.002, 0.002 | 0.93 |
| shoot biomass fraction vs. total nitrogen | 80-100 | 0.00 | 0.00 | -0.002, 0.003 | 0.90 |
| shoot biomass fraction vs. total nitrogen | 100-150 | 0.00 | 0.00 | -0.003, 0.001 | 0.35 |
| shoot biomass fraction vs. total nitrogen | 150-200 | 0.00 | 0.00 | -0.004, 0.001 | 0.26 |
