# Supplementary material for: Effects of biotic and abiotic factors on forest biomass fractions
Source: Natl Sci Rev. 2021 Apr 2;8(10):nwab025. doi: 10.1093/nsr/nwab025 (PMC8566188; doi:10.1093/nsr/nwab025)
Supplement: nwab025_Online_Appendixs [file nwab025_online_appendixs.zip › AppendixC.docx]

**Effects of climate on NPP and biomass for different organ compartments**

In general, climate (MAT or MAP) had a relatively strong effect on plant NPP for the different organ compartments but had a relatively weak effect on plant NPP fractions (table S7, figure S3). The regression slopes for leaf NPP vs. MAP, aboveground NPP vs. MAP, belowground NPP vs. MAP, and total NPP vs. MAP were 0.50 (*r*^2^ = 0.04, *P*<0.0001, *n* = 418), 0.73 (*r*^2^ = 0.14, *P*<0.0001, *n* = 418), 0.95 (*r*^2^ = 0.12, *P*<0.0001, *n* = 418), and 0.73 (*r*^2^ = 0.14, *P*<0.0001, *n* = 418), respectively. The slopes for the NPP of the different organ compartments vs. MAT were 14.34 (*r*^2^ = 0.10, *P*<0.0001, *n* = 418), 18.25 (*r*^2^ = 0.25, *P*<0.0001, *n* = 418), 22.57 (*r*^2^ = 0.19, *P*<0.0001, *n* = 418), and 18.25 (*r*^2^ = 0.25, *P*<0.0001, *n* = 418), respectively (table S7, figure S3). However, the regression slopes of leaf NPP fraction vs. MAP and shoot NPP fraction vs. MAP were -0.23 (*r*^2^ = 0.02, *P*=0.01, *n* = 418) and -0.001 (*r*^2^ < 0.0001, *P* = 0.95, *n* = 418), respectively, and they were -3.91 (*r*^2^ = 0.02, *P* = 0.01, *n* = 418) and -0.0002 (*r*^2^ < 0.0001, *P =* 0.99, *n* = 418), respectively, for NPP fractions vs. MAT(table S7, figure S3).

In comparison with NPP, climate had a much weaker effect on plant biomass for the different plant organ compartments (table S7, figure S6). The regression slopes of leaf biomass vs. MAP, total biomass vs. MAP, aboveground biomass vs. MAP, and belowground biomass vs. MAP were 0.27 (*r*^2^ = 0.01, *P =* 0.00, *n* = 2347), 0.04 (*r*^2^ < 0.001, *P =* 0.59, *n* = 2347), -0.05 (*r*^2^ < 0.001, *P =* 0.50, *n* = 1349), and -0.03 (*r*^2^ < 0.001, *P =* 0.67, *n* = 1349), respectively. The slopes for the relationships of the corresponding variables vs. MAT were 1.95 (*r*^2^ < 0.001, *P =* 0.06, *n* = 2347), -4.06 (*r*^2^ = 0.00, *P =* 0.00, *n* = 2347), -4.96 (*r*^2^ = 0.01, *P =* 0.00, *n* = 1349), and -4.51 (*r*^2^ = 0.01, *P =* 0.00, *n* = 1349), respectively (table S7, figure S6). However, climate had a much stronger effect on plant biomass when the data set was separated into different plant age classes (table S8, figure S6).

**Why climate has a strong effect on NPP but a weak effect on biomass**

Our analyses show that NPP is much more sensitive than biomass to climate (MAP or MAT). This observation indicates a difference between NPP and standing biomass, and a difference between plant size and age. NPP reflects annual biomass accumulation, whereas the standing biomass of a plant reflects the accumulation of biomass throughout a plant’s lifespan. Thus, the effect of climate on NPP can be revealed immediately through simple bivariate regression analysis, whereas the effect of climate on standing biomass is time-averaged over plant age. Although plant size and age have a strong relationship, they cannot be used as substitutes for each other when investigating the effect of climate on plant growth. A plant’s growth rate will decrease under harsh environmental conditions, such as drought or cold. As a consequence, more time is required to achieve the same body size as a conspecific growing under more equitable conditions. Therefore, the effect of climate on plant biomass can be observed only when plants are sorted into comparable age classes. To test this hypothesis, we separated our data sets into different age classes, which revealed that both MAT and MAP generally have a significant and strong effect on plant biomass (leaf biomass, total biomass, aboveground biomass and belowground biomass) within different age classes regardless of plant age (table S8, figure S6). These results caution against using a single plant size vs. age scaling relationship when studying the effect of climate on plant biomass and NPP allocation patterns among different organ compartments.

Although climate has a strong effect on NPP for different organ compartments, its effects on NPP fractions (leaf NPP fraction and shoot NPP fraction) are much weaker (figure S3). This difference reveals one of the characteristics of fraction values: the climate effect is cancelled out in the quotient of the denominator and the numerator. This finding suggests to us that climate has an equivalent effect on the NPP of different plant organ compartments. The equivalent climate effect on different plant organ compartments provides an additional reason why climate has little effect on biomass fractions (figures 2, S2, 3).
